# Tetrahydrobiopterin metabolism attenuates ROS generation and radiosensitivity through LDHA S-nitrosylation: novel insight into radiogenic lung injury

**DOI:** 10.1038/s12276-024-01208-z

**Published:** 2024-05-01

**Authors:** Yang Feng, Yahui Feng, Liming Gu, Wei Mo, Xi Wang, Bin Song, Min Hong, Fenghao Geng, Pei Huang, Hongying Yang, Wei Zhu, Yang Jiao, Qi Zhang, Wei-Qun Ding, Jianping Cao, Shuyu Zhang

**Affiliations:** 1 State Key Laboratory of Radiation Medicine and Protection, School of Radiation Medicine and Protection, Medical College of Soochow University, 215123 Suzhou, China; 2 Department of Oncology, Wuxi No.2 People’s Hospital, Jiangnan University Medical Center, 214002 Wuxi, China; 3 Laboratory of Radiation Medicine, Second Affiliated Hospital of Chengdu Medical College, China National Nuclear Corporation 416 Hospital, 610051 Chengdu, China; 4 West China Second University Hospital, Sichuan University, 610041 Chengdu, China; 5 Department of Pathology, Stephenson Cancer Centre, College of Medicine, University of Oklahoma Health Sciences Center, Oklahoma City, OK 73104 USA; 6 Laboratory of Radiation Medicine, West China School of Basic Medical Sciences & Forensic Medicine, Sichuan University, 610041 Chengdu, China; 7 NHC Key Laboratory of Nuclear Technology Medical Transformation (Mianyang Central Hospital), 621099 Mianyang, China

**Keywords:** Post-translational modifications, Molecular biology

## Abstract

Genotoxic therapy triggers reactive oxygen species (ROS) production and oxidative tissue injury. S-nitrosylation is a selective and reversible posttranslational modification of protein thiols by nitric oxide (NO), and 5,6,7,8-tetrahydrobiopterin (BH4) is an essential cofactor for NO synthesis. However, the mechanism by which BH4 affects protein S-nitrosylation and ROS generation has not been determined. Here, we showed that ionizing radiation disrupted the structural integrity of BH4 and downregulated GTP cyclohydrolase I (GCH1), which is the rate-limiting enzyme in BH4 biosynthesis, resulting in deficiency in overall protein S-nitrosylation. GCH1-mediated BH4 synthesis significantly reduced radiation-induced ROS production and fueled the global protein S-nitrosylation that was disrupted by radiation. Likewise, *GCH1* overexpression or the administration of exogenous BH4 protected against radiation-induced oxidative injury in vitro and in vivo. Conditional pulmonary *Gch1* knockout in mice (*Gch1*^*fl/fl*^; *Sftpa1-Cre*^*+/−*^ mice) aggravated lung injury following irradiation, whereas *Gch1* knock-in mice (*Gch1*^*lsl/lsl*^; *Sftpa1-Cre*^*+/−*^ mice) exhibited attenuated radiation-induced pulmonary toxicity. Mechanistically, lactate dehydrogenase (LDHA) mediated ROS generation downstream of the BH4/NO axis, as determined by iodoacetyl tandem mass tag (iodoTMT)-based protein quantification. Notably, S-nitrosylation of LDHA at Cys163 and Cys293 was regulated by BH4 availability and could restrict ROS generation. The loss of S-nitrosylation in LDHA after irradiation increased radiosensitivity. Overall, the results of the present study showed that GCH1-mediated BH4 biosynthesis played a key role in the ROS cascade and radiosensitivity through LDHA S-nitrosylation, identifying novel therapeutic strategies for the treatment of radiation-induced lung injury.

## Radiation-induced Lung Injury Mitigated by GCH1-Mediated ROS Regulation

Radiation therapy for cancer can harm healthy tissues, causing swelling and oxidative stress (an imbalance between free radicals and antioxidants in your body). This research examined the part of a molecule named tetrahydrobiopterin (BH4) in this. The scientists discovered that radiation therapy decreases the amount of BH4 in the body, which then leads to a rise in harmful reactive oxygen species (ROS - molecules that can damage cells). However, when BH4 amounts were artificially boosted, this lowered ROS levels and shielded against radiation-caused harm. This implies that BH4 might be used as a treatment to guard against the damaging side effects of radiation therapy. More research is required to further investigate this potential in clinic.

This summary was initially drafted using artificial intelligence, then revised and fact-checked by the author.

## Introduction

Each year, millions of cancer patients undergo genotoxic therapy to treat malignant masses surrounded by noncancerous tissues or normal cell environments^1,2^. Genotoxic therapy, such as radiation, induces oxidative damage and inflammatory responses in normal tissues^3,4^. The most critical mediators of intracellular signals involved in radiation-induced toxicity are free radicals, such as reactive oxygen species (ROS), which can lead to oxidative damage of DNA, proteins and lipids. Alterations in the normal functions of reduction/oxidation (redox) systems can amplify free radical production following exposure to radiation^5^. Depending on the tissue/cell type and quality of ionizing radiation, these signals can persist for hours, days or months after exposure, which leads to early and late tissue injury^6,7^. Identifying the mechanisms by which ROS affect these signals is important for clarifying tissue radiosensitivity and protecting against oxidative injury by targeting mediators.

**Fig. 1:**
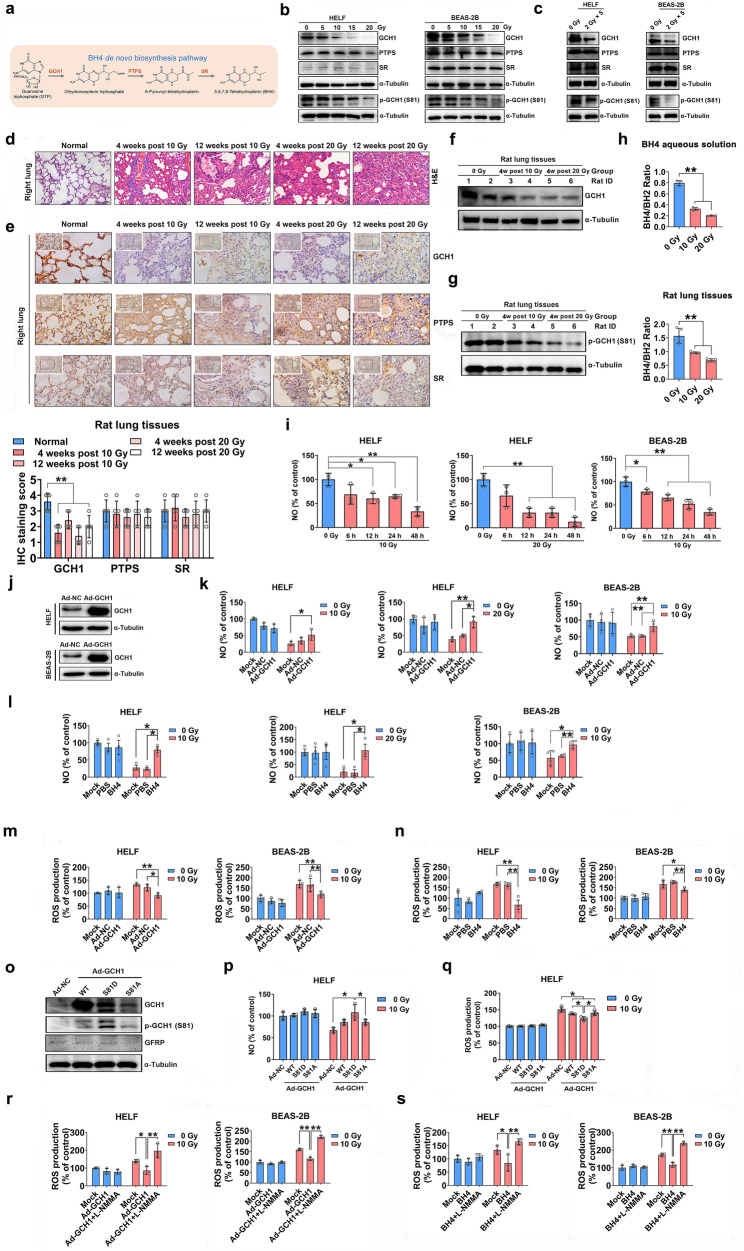
BH4 metabolism attenuates radiation-induced ROS generation by reducing NOS uncoupling and restoring NO levels. **a** Schematic illustration of the BH4 de novo biosynthesis pathway. **b** Western blot analysis of GCH1, PTPS and SR expression and GCH1 phosphorylation in HELF and BEAS-2B cells 24 h after the indicated doses of radiation. **c** Western blot analysis of GCH1, PTPS and SR expression and GCH1 phosphorylation in HELF and BEAS-2B cells exposed to radiation (10 Gy/5 fractions, 1 fraction/day). **d** Representative H&E staining of rat lungs. Scale bar = 100 μm. **e** Immunohistochemistry was used to measure GCH1, PTPS and SR levels in irradiated lung tissues. Scale bar = 50 μm. Quantitative analysis of GCH1-, PTPS- and SR-positive cells in rat lung tissues. **f** Western blot analysis of GCH1 protein levels in rat lung tissues. **g** GCH1 phosphorylation in rat lung tissues. **h** The BH4/BH2 ratio in BH4 aqueous solutions (1 μg/L) and rat lung tissues exposed to 0, 10, or 20 Gy of ionizing radiation. **i** NO concentrations at different time points after irradiation were measured using an NO-sensitive probe. **j** Western blot analysis of GCH1 expression in HELF and BEAS-2B cells. **k** HELF and BEAS-2B cells were infected with the control adenovirus or GCH1 adenovirus and then exposed to 10 Gy of X-ray irradiation. NO concentrations were measured using an NO-sensitive probe. **l** The cells were incubated with BH4 (12.5 μM for HELF cells and 25 μM for BEAS-2B cells) or PBS followed by exposure to 10 Gy of X-ray irradiation. NO concentrations were measured with an NO fluorescent probe. **m** and **n** ROS levels in HELF and BEAS-2B cells were determined using a fluorescence microscope and 96-well plate reader. **o** Western blot analysis of GCH1 and GFRP expression and GCH1 phosphorylation in HELF cells. HELF cells that were transfected with plasmids encoding WT GCH1 or its mutants. **p** NO concentrations were measured using an NO-sensitive probe. **q** ROS levels in HELF cells were determined using a 96-well plate reader. **r** HELF and BEAS-2B cells were incubated with the NOS inhibitor L-NMMA (200 μM for HELF cells and 100 μM for BEAS-2B cells) for 6 h and then infected with the GCH1 adenovirus followed by exposure to 10 Gy of X-ray irradiation. ROS levels in HELF and BEAS-2B cells were determined using a 96-well plate reader. **s** HELF and BEAS-2B cells were incubated with the NOS inhibitor L-NMMA for 6 h and then treated with BH4. ROS levels in HELF and BEAS-2B cells were measured using a 96-well plate reader. *P < 0.05 and **P < 0.01 compared with the control group.

5,6,7,8-Tetrahydrobiopterin (BH4) is an essential cofactor for multiple enzymes, including nitric oxide synthase (NOS) and aromatic amino acid hydroxylases^8,9^. BH4 is synthesized by two pathways: a de novo pathway and a salvage pathway^8^. De novo BH4 synthesis refers to the process by which GTP undergoes a series of enzymatic reactions to form BH4 and is the main BH4 synthesis pathway. Under normal physiological conditions, the biosynthesis of BH4 from GTP is carried out by three enzymes: GTP cyclohydrolase I (GCH1), 6-pyruvoyltetrahydropterin synthase (PTPS), and sepiapterin reductase (SR) (Fig. 1a). Loss of function of any of these enzymes reduces BH4 production and uncouples nitric oxide (NO) generation from L-arginine oxidation, resulting in the production of highly oxidative radicals, including superoxide and peroxynitrite^9^. BH4 is reductive and easily oxidized to dihydrobiopterin (BH2) when damaged. BH2 causes NOS uncoupling to produce ROS^10,11^. In addition, BH4 homeostasis has been implicated in ferroptosis, which is an iron-dependent form of regulated cell death^12^. BH4 has thus been implicated in radiation adaptation and the modulation of radiation-induced injury^13–15^. However, the mechanism by which BH4 regulates radiosensitivity is not well understood.

NOSs has three isoforms and normally catalyzes the production of NO and L-citrulline from L-arginine in the presence of sufficient BH4^16^. As a lipophilic messenger molecule, NO participates in various physiological and pathological activities, including inflammation, immune regulation, vasodilation and angiogenesis^17,18^. The ubiquitous effects of NO on cellular signaling can also be mediated by the S-nitrosylation of protein cysteine residues^19,20^. S-nitrosylation is a ubiquitous redox-related modification of cysteine thiols by NO and involves the addition of a thiyl radical-linked NO or NO-derived nitrosating species to the reduced thiol of a cysteine residue^21^. S-nitrosylation is involved in the regulation of gene transcription^22^, enzyme activity^23^ and nuclear protein translocation^19^.

However, the role of BH4 metabolism in protein S-nitrosylation and its relationship with the amplification of ROS remain unclear. In this study, we characterized the role of GCH1-mediated BH4 anabolism by examining radiation-induced ROS production and protein S-nitrosylation. Our findings revealed that the GCH1/BH4 axis played a novel and critical role in radiation-induced ROS amplification through LDHA S-nitrosylation, providing therapeutic strategies to protect against radiation-induced lung injury.

## Materials and methods

### Cell culture and irradiation

Human embryonic lung fibroblasts HELF cells and human pulmonary epithelial BEAS-2B cells were used as previously reported^24,25^. The cells were maintained in high-glucose Dulbecco’s modified Eagle medium (HyClone, Logan, UT) supplemented with 10% fetal bovine serum (Gibco, Grand Island, NY) and incubated at 37 °C in a humidified atmosphere containing 5% CO_2_. The cells were exposed to 10 or 20 Gy of ionizing radiation using an X-ray linear accelerator (RadSource, Suwanee, GA) at a fixed dose rate of 1.15 Gy/min.

### ROS generation assay

ROS levels were determined using the ROS-sensitive dye 2,7-dichlorofluorescein diacetate (DCFH-DA; Nanjing Jiancheng Bioengineering Institute, Nanjing, China). Briefly, cells were incubated with 10 μΜ DCFH-DA for 30 min in the dark, washed with PBS, and observed under a fluorescence microscope (Leica IX73, Hessian, Germany). Lung tissues were trypsinized to prepare single-cell suspensions according to the manufacturer’s instructions. For quantification, the level of 2,7-dichlorofluorescein fluorescence in cells and lung tissues was measured using a 96-well plate reader (Ex/Em = 488 nm/525 nm).

### NO determination

NO concentrations were determined with an NO-sensitive fluorescence probe (Beyotime, Nantong, China) according to the manufacturer’s instructions. Briefly, the cells were incubated with DAF-FM DA for 20 min in the dark and washed with PBS, after which the fluorescence intensity of DAF-FM DA was measured by a 96-well plate reader (Ex/Em = 495 nm/515 nm).

The concentrations of nitrate and nitrite were measured by a modified Griess reaction method to indirectly measure total NO concentrations in the cultured cells. After the cells were lysed, NO was measured using a total NO assay kit (Beyotime). For lung tissues, NO concentrations were measured at 540 nm by a 96-well plate reader.

### Lactate dehydrogenase release assay

Cell death was measured by determining the amount of lactate dehydrogenase (LDH) released into the supernatant using an LDH cytotoxicity assay kit (Beyotime, Nantong, China) according to the manufacturer’s instructions. The absorbance was measured at 490 nm using a microplate reader (BioTek, Winooski, VT), and the results are presented as the means ± SEMs.

### Mice and irradiation

Protocols for experiments involving mice were approved by the Animal Experimentation Ethics Committee of Soochow University (Suzhou, China) and Sichuan University (Chengdu, China). Six- to eight-week-old control (*Gch1*^*fl/fl*^; *Sftpa1-Cre*^*−/−*^) and *GCH1* conditional KO (*Gch1*^*fl/fl*^; *Sftpa1-Cre*^*+/−*^) male mice and control (*Gch1*^*lsl/lsl*^; *Sftpa1-Cre*^*−/−*^) and conditional knock-in (*Gch1*^*lsl/lsl*^; *Sftpa1-Cre*^*+/−*^) male mice were generated by GemPharmatech (Nanjing, China). The mice were housed under a 12-h light/dark cycle and had free access to food and water. For the loss-of-function study, WT mice and *Gch1*-CKO mice were randomly divided into two groups: 1) the nonirradiated control group (control and *Gch1*-CKO) and 2) the irradiated control group (control and *Gch1*-CKO). Mice in the irradiated group was administered a single dose of 15 Gy radiation to the thorax using an X-ray linear accelerator (RadSource, Suwanee, GA). The mice were sacrificed, and samples were collected on Days 7 and 90 after irradiation. For the gain-of-function study, control and *Gch1*-CKI mice received the same treatment as described above.

Male C57BL/6 mice aged 6-8 weeks and weighing 22–25 g were purchased from Shanghai SLAC Laboratory Animal Co., Ltd. (Shanghai, China). A total of 25 mice were randomly divided into five groups (*n* = 5): (1) the nonirradiated control group; (2) the irradiated group injected with Ad-NC; (3) the irradiated group injected with Ad-GCH1; (4) the irradiated group administered PBS; and (5) the irradiated group administered BH4. A single dose of 20 Gy irradiation was administered to the right lung area using a small animal radiotherapy simulation localization instrument with an aperture of 5 mm (X-RAD SmART, Precision X-ray, Inc., Branford, CT). Mice in the irradiation groups were administered 100 μL of Ad-NC (1.0 × 10^12^ vp/mL), Ad-GCH1 (1.0 × 10^12^ vp/mL), PBS or BH4 (1 mg/kg) via the tail vein. The mice were sacrificed, and tissue samples were collected on Days 7 and 90 after irradiation.

### Hematoxylin and eosin staining

Lung tissues were fixed in 10% neutral-buffered formalin and embedded in paraffin. In total, 3-µ paraffin sections were deparaffinized and heated with citrate buffer (pH = 6.0) for seven minutes according to an epitope retrieval protocol. The lung sections were stained with Hematoxylin and eosin (H&E).

### Masson’s trichrome staining

In total, 3-µ-thick slices were cut from the lung tissue blocks in the experimental groups and incubated with xylene (2 × 5 min) and a descending alcohol series (100%, 90%, 80%, 70% and 50%) for deparaffinization. Next, a Masson’s Trichrome Stain Kit (Solarbio, Beijing, China) was used to analyze the sections.

### Detection of S-nitrosylation by a biotin switch assay

A biotin switch assay (Abcam, #ab236207, Cambridge, MA) was used to directly visualize S-NO proteins in HELF cells. Free SH groups were first blocked, after which the S-NO bonds present in HELF cells were cleaved. Biotinylation of the newly formed SH groups and streptavidin-based colorimetric detection were performed for visualization. Briefly, the cells were homogenized in S-nitrosylation wash buffer. After centrifugation, S-nitrosylation buffer A containing blocking reagent was added to the supernatant and incubated at 4 °C for 30 min to block free thiols. After acetone precipitation and centrifugation, the pellet was resuspended in S-nitrosylation buffer B containing reducing and labeling reagents and incubated at room temperature for 1 h. Then, excess Buffer B was removed by ice-cold acetone precipitation. The protein pellet was resuspended in cold S-nitrosylation wash buffer. The samples were analyzed by Western blotting to examine total S-nitrosylated proteins or analyzed by immunoprecipitation to examine S-nitrosylated LDHA.

### LDH enzymatic activity assay

Cell and tissue supernatants were collected, and LDH activity was measured. Briefly, 50 μL of the standard, cell or tissue sample or positive control sample was loaded into a 96-well plate, after which 50 μL of the reaction mixture (LDH assay buffer or LDH substrate mixture) was added to each well. After being mixed, the sample was immediately measured at OD 450 nm on a microplate reader in kinetic mode. LDH activity was then calculated according to the manufacturer’s instructions.

### Statistical analysis

The data are expressed as the mean ± SEM of at least three independent experiments. Multiple treatments were analyzed by one-way ANOVA followed by Tukey’s test for multiple comparisons. Student’s *t test* was used to compare two groups to determine statistical significance. Survival curves were assessed based on the Kaplan–Meier method and compared using the log-rank test. The statistical analyses were performed using Prism 8 software (GraphPad Software, La Jolla, CA). Differences were considered significant at *P* < 0.05 (other methods are detailed in the Supplementary Materials and Methods).

## Results

### Ionizing radiation decreases GCH1 expression and reduces BH4 availability, which impairs NO homeostasis

To determine the effects of ionizing radiation on BH4-associated metabolic pathways, the expression of GCH1, PTPS, and SR, which are enzymes involved in the de novo BH4 synthase pathway (Fig. 1a), was measured in cell and animal models by Western blot analysis. Radiation decreased the expression of GCH1 but not PTPS or SR in a dose-dependent manner in HELF and BEAS-2B cells (Fig. 1b and Supplementary Fig. 1a, b), indicating that the synthesis of BH4 was suppressed in a dose-dependent manner after irradiation. To simulate radiotherapy, pulmonary cells were treated with radiation (10 Gy/5 fractions, 1 fraction/day)^26^; fractionated irradiation reduced GCH1 expression but not PTPS or SR expression (Fig. 1c and Supplementary Fig. 1c). A rat model of radiation-induced lung injury was established^27^, and morphological changes associated with lung injury and fibrosis were visualized by H&E staining (Fig. 1d). The radiation doses were selected to match the relatively larger fractional doses delivered during radiotherapy, such as stereotactic body radiation therapy (SBRT)^28,29^. GCH1 expression in irradiated rat lung tissues was lower than that in nonirradiated rats and was partially restored 12 weeks postirradiation, as determined by immunohistochemistry and Western blot analysis (Figs. 1e, f). However, there were no significant changes in PTPS or SR protein levels in any of the groups (Fig. 1e). The decrease in GCH1 expression in response to radiation was partially attributed to the ubiquitin proteasome pathway (Supplementary Fig. 1d).

A phosphorylation site (serine 81, S81) in the human GCH1 protein mediates the significant increase in BH4 production, thereby increasing NO production^30^. This resistance is partially due to resistance to feedback inhibition by GTP cyclohydrolase feedback regulatory protein (GFRP), an essential modulator of GCH1 enzyme activity^30^. As expected, ionizing radiation decreased GCH1 phosphorylation in a dose-dependent manner in HELF and BEAS-2B cells (Fig. 1b). Similarly, GCH1 phosphorylation was reduced after fractional irradiation (Fig. 1c). Western blot analysis revealed a radiation-induced decrease in GCH1 phosphorylation compared with that in unirradiated rat lung tissue (Fig. 1g).

As an important cofactor for NOS, BH4 is easily oxidized to various derivatives, such as BH2, even in response to UV irradiation^31,32^. As expected, the BH4/BH2 ratio in an aqueous solution of BH4 or in rat lung tissues decreased significantly after ionizing radiation (Fig. 1h). We subsequently measured cellular NOS activity, and irradiation decreased NOS activity in HELF and BEAS-2B cells (Supplementary Fig. 1e). Furthermore, Western blotting was performed to examine the protein levels of each NOS isoform. All three NOS isoforms were expressed in HELF and BEAS-2B cells. Importantly, irradiation decreased eNOS protein levels but not iNOS or nNOS protein levels in a time-dependent manner (Supplementary Fig. 1f). Next, NO levels were measured with an NO-sensitive probe (DAF-FM DA) and the Griess assay based on NaNO_2_ levels. NO levels were significantly lower in irradiated HELF and BEAS-2B cells than in nonirradiated cells (Fig. 1i and Supplementary Fig. 1g). Consistent with this finding, GCH1 was slightly reduced at 6 h after irradiation, while at 12 h after irradiation, GCH1 was significantly reduced (Supplementary Fig. 1h), indicating that the decrease in NO levels was mediated by the loss of GCH1 expression. These results indicated that ionizing radiation disrupted the de novo synthesis of BH4, thereby disturbing NO homeostasis.

### BH4 biosynthesis decreases eNOS uncoupling

A lack of BH4 leads to NOS uncoupling and the production of ROS by NOS^31^. We therefore investigated whether radiation-induced ROS production was regulated by GCH1 or its metabolite BH4. Infection with a *GCH1*-overexpressing adenovirus (Ad-GCH1) significantly increased GCH1 protein levels in HELF and BEAS-2B cells (Fig. 1j). We then measured cellular NO levels with NO fluorescent probes. The results showed that Ad-GCH1 infection did not affect cellular NO levels in HELF or BEAS-2B cells in the absence of radiation (Fig. 1k). Although NO levels in Ad-GCH1-infected HELF after 10 Gy irradiation was not significantly increased (Fig. 1k, *P* = 0.14), *GCH1* overexpression significantly increased NO production in HELF cells irradiated with 20 Gy. These results suggested that *GCH1* overexpression restored NO levels in irradiated lung fibroblasts. Exogenous BH4 can penetrate the cell membrane^33^. We therefore pretreated HELF and BEAS-2B cells with BH4 prior to irradiation. As shown in Fig. 1l, BH4 reversed the decrease in NO concentrations caused by radiation. Because BH4 is involved in NO/ROS homeostasis, we then examined the effect of GCH1 on intracellular ROS levels following irradiation. Overexpression of *GCH1* did not change ROS levels in the absence of radiation (Fig. 1m and Supplementary Fig. 2a, b), whereas the level of ROS in the Ad-GCH1 group was significantly reduced following exposure to 10 or 20 Gy of radiation (Fig. 1m and Supplementary Fig. 2a, b). Similarly, the addition of BH4 reduced the ionizing radiation-induced increases in ROS levels in HELF and BEAS-2B cells (Fig. 1n and Supplementary Fig. 2c, d). Thus, our results demonstrated that the GCH1/BH4 axis was responsible for cellular NO/ROS homeostasis following irradiation.

The effect of GCH1 phosphorylation at serine 81 (S81) on radiation-induced ROS production has not been determined. Therefore, HELF cells were preinfected with adenoviruses encoding wild-type (WT) GCH1 or mutant GCH1 in which S81 was replaced with an aspartate to mimic phosphorylation (Ad-GCH1-S81D) or an alanine to block phosphorylation (Ad-GCH1-S81A). As expected, infection with WT GCH1 or the mutants induced GCH1 expression via differential phosphorylation (Fig. 1o), which did not significantly affect GFRP expression (Fig. 1o). Irradiated HELF cells infected with the GCH1 S81D mutant (Ad-GCH1-S81D) produced significantly more NO and less ROS than those infected with WT GCH1 (Ad-GCH1) or the S81A mutant (Ad-GCH1-S81A) (Figs. 1p, q). These results indicated that GCH1 S81 phosphorylation was critical for radiation-induced ROS production.

BH4 serves as an essential cofactor for NOS and several aromatic amino acid hydroxylases^8,9^. We next investigated whether the GCH1/BH4 axis modulated radiogenic ROS by inhibiting NOS uncoupling. We found that the elimination of radiation-induced ROS by *GCH1* overexpression was abrogated by the NOS inhibitor L-NMMA in HELF cells and BEAS-2B cells (Fig. 1r and Supplementary Fig. 3a, b). Consistently, L-NMMA treatment abolished the reduction in radiation-induced ROS induced by BH4 supplementation (Fig. 1s and Supplementary Fig. 3c, d). In addition, superoxide anion concentrations in the two cell lines were determined with a dihydroethidium (DHE)-based fluorescent probe. The results showed that irradiation significantly increased superoxide anion generation. However, the production of superoxide anions was reduced after GCH1/BH4 treatment, and the decrease in radiation-induced superoxide anions induced by GCH1/BH4 treatment could be reversed by L-NMMA pretreatment (Supplementary Fig. 3e, f). Furthermore, L-NAME, which is another NOS inhibitor, was used to validate these results. Consistently, L-NAME treatment also abolished the reduction in radiation-induced ROS and superoxide anion levels induced by *GCH1* overexpression or BH4 supplementation (Supplementary Fig. 4a–d). These results indicated that NOS activity was essential for the attenuation of radiogenic ROS production mediated by GCH1 or its metabolite BH4.

### BH4 metabolism protects against radiation-induced damage in vitro

**Fig. 2:**
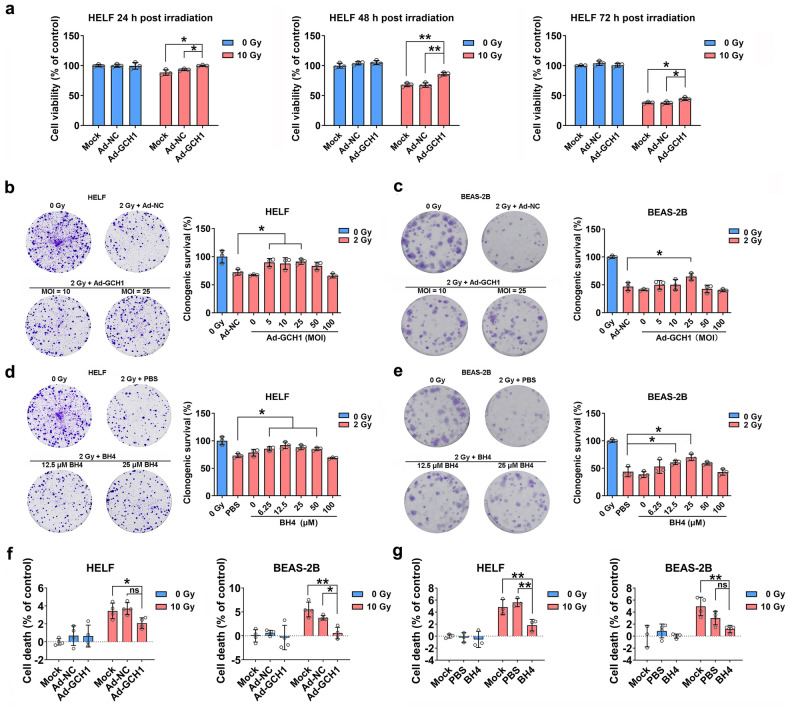
BH4 metabolism protects pulmonary cells from radiation-induced damage. **a** HELF cells were infected with the control adenovirus or GCH1 adenovirus and subsequently irradiated. Cell viability was measured by a CCK-8 assay at 24, 48, and 72 h after irradiation. The effect of *GCH1* overexpression on the survival of (**b**) HELF and (**c**) BEAS-2B cells was evaluated. HELF and BEAS-2B cells were treated with BH4 or PBS and subsequently irradiated. **d**, **e** The clonogenic survival of the two cell lines was measured. **f**, **g** HELF and BEAS-2B cells were subjected to LDH release assays. *P < 0.05 and **P < 0.01 compared with the control group.

To determine the effect of GCH1 on radiation-induced damage, human embryonic lung fibroblasts and human pulmonary epithelial cells were infected with Ad-NC or Ad-GCH1 and subsequently irradiated. After 24, 48, or 72 h, cell viability in each group was measured by a CCK-8-based assay. Ad-GCH1 did not affect the viability of HELF cells in the unirradiated group (Fig. 2a and Supplementary Fig. 5a). However, the viability of cells in the Ad-GCH1 group was significantly higher than that of control adenovirus-infected cells after 10 or 20 Gy irradiation (Fig. 2a and Supplementary Fig. 5a). Consistent with these findings, BH4 treatment increased the viability of irradiated lung cells (Supplementary Fig. 5b). The effect of GCH1/BH4 on cellular radiosensitivity and survival was further determined by a clonogenic survival assay. Overexpression of *GCH1* significantly increased the survival of HELF and BEAS-2B cells following irradiation (the highest survival occurred at an MOI of 25:1; Fig. 2b, c). Consistently, BH4 increased the survival of the two cell lines (Fig. 2d, e). To further measure the effect of the GCH1/BH4 axis on cellular damage, LDH release was analyzed. As shown in Fig. 2f, the percentage of death in irradiated HELF and BEAS-2B cells was significantly decreased after infection with Ad-GCH1. Similarly, BH4 reduced death in the two irradiated cell lines (Fig. 2g). Taken together, these results indicated that overexpression of *GCH1* significantly alleviated cellular damage and enhanced cell survival after exposure to ionizing radiation.

### BH4 metabolism modulates the fibrotic phenotypes of irradiated pulmonary cells

Pulmonary fibrosis is the most common outcome of radiation-induced lung injury^34^. TGF-β signaling plays important roles in various fibrotic disorders, including radiation-induced fibrosis^35^. To further explore the role of GCH1 in the activity of the TGF-β/Smad pathway, a Smad2/3-responsive luciferase reporter was generated as previously reported^35^. *N*-Acetyl-l-cysteine (NAC), an oxygen free-radical scavenger, is able to suppress TGF-β signaling^36^. a positive control^36^, NAC reduced the luciferase activity of the Smad2/3-responsive reporter in HELF cells (Supplementary Fig. 6a, b). Infection with Ad-GCH1 or the addition of BH4 significantly decreased the luciferase activity of the Smad2/3-responsive reporter following radiation (Supplementary Fig. 6a, b). As shown in Fig. 1, GCH1/BH4 increased the production of NO, which is a known inhibitor of TGF-β signaling^37^, suggesting that GCH1/BH4/NO reduced TGF-β signaling in lung fibroblasts.

Since GCH1 expression can be restored 12 weeks after irradiation (Fig. 1d) and occurs within a fibrotic microenvironment, we investigated the relationship between GCH1 and the pulmonary fibrotic phenotype. HELF cells were transfected with siNC or siGCH1, and *GCH1* silencing significantly decreased GCH1 protein levels (Supplementary Fig. 6c). As expected, ROS levels were increased after *GCH1* was silenced (Supplementary Fig. 6d). After 20 Gy of irradiation and/or TGF-β1 stimulation, *GCH1* knockdown further increased intracellular ROS levels (Supplementary Fig. 6d). Smad2/3 is phosphorylated in response to TGF-β1 stimulation, and activated Smad2/3 binds with Smad4 to form a complex that carries the signal into the nucleus, thereby promoting collagen synthesis, inhibiting its degradation, and eventually leading to fibrosis^38^. Immunofluorescence assays also revealed that the distribution of Smad2 in the nucleus was increased by the addition of exogenous TGF-β1 and/or X-ray exposure (Supplementary Fig. 6e). Intriguingly, *GCH1* knockdown further increased the nuclear translocation of Smad2 and significantly increased the luciferase activity of the Smad2/3-responsive luciferase reporter in HELF cells (Supplementary Fig. 6e, f). These results indicated that the attenuation of BH4 metabolism promotes the development of a fibrotic phenotype.

### *GCH1* modulates radiation-induced acute lung injury in mice

**Fig. 3:**
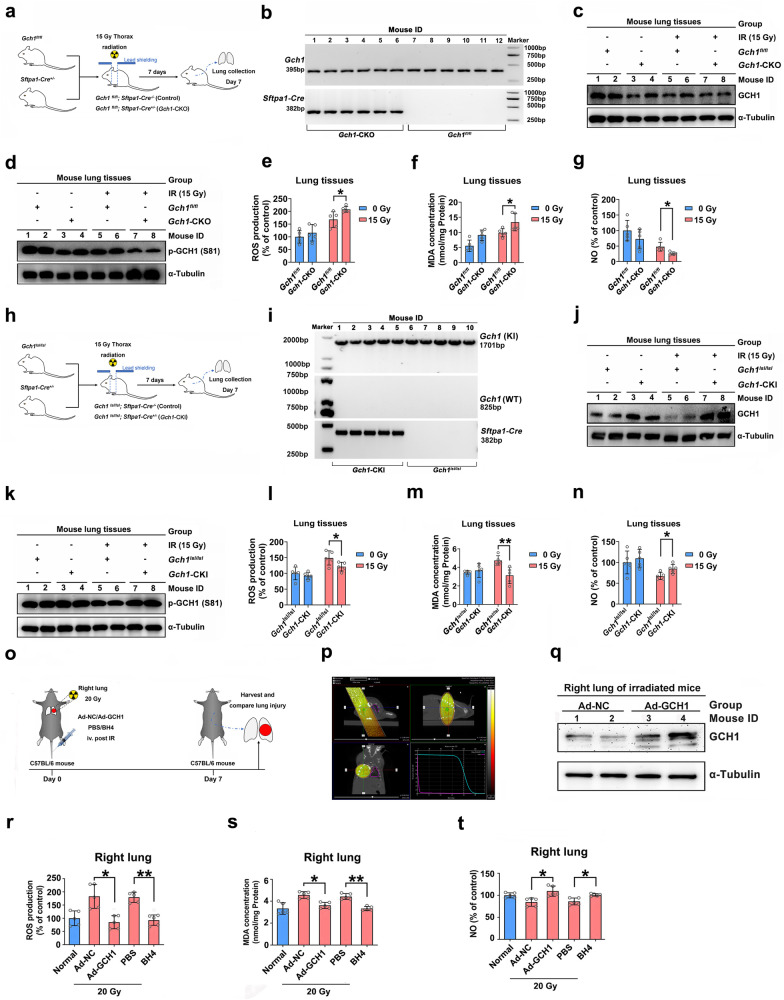
BH4 anabolism attenuates acute radiation-induced lung injury. **a** Experimental scheme for the treatment of *Gch1*-CKO mice. **b** Identification of mouse genotypes. **c** Western blot analysis of GCH1 expression in the lung tissues of *Gch1*-CKO mice. **d** Western blot analysis of GCH1 phosphorylation in lung tissues. **e** MDA levels in the lung tissues of mice in the various groups. **f** Seven days after irradiation, relative NO levels in mouse lungs were determined in each group. **g** Western blot analysis of TFR1, FTH1, HO-1, and GPX4 expression in the lung tissues of *Gch1*-CKO mice. **h** Experimental scheme for the treatment of *Gch1*-CKI mice. **i** Identification of mouse genotypes. **j** Western blot analysis of GCH1 expression in the lung tissues of *Gch1*-CKI mice. **k** Western blot analysis of GCH1 phosphorylation in lung tissues. **l** MDA levels in the lung tissues of *Gch1*-CKI mice. **m** Relative NO levels in mouse lungs. **n** Western blot analysis of TFR1, FTH1, HO-1, and GPX4 expression in the lung tissues of *Gch1*-CKI mice. **o** Experimental scheme for the treatment of mice. The right lung was not irradiated or was irradiated with a single dose of 20 Gy of X-ray irradiation followed by i.v. injection of Ad-NC or Ad-GCH1 and PBS or BH4 (*n* = 5). **p** Target volume determination of the mice. **q** Western blot analysis of GCH1 expression in the two groups of mice. **r** Relative ROS levels in the right lung. **s** MDA levels in the right lung tissues of the mice in the various groups. **t** NO levels in the right lung were determined in each group. *P < 0.05 and **P < 0.01 compared with the control group.

Animal models of radiation-induced lung injury, including acute injury and fibrosis, were used for subsequent in vivo studies. Previous studies have shown that *Gch1*-deficient mice exhibit bradycardia and embryonic death during midgestation^39^. We constructed control mice (*Gch1*^*fl/fl*^ mice) and pulmonary-specific *Gch1* knockout (KO) mice (*Gch1*^*fl/fl*^; *Sftpa1-Cre*^*+/−*^, *Gch1*-CKO mice), and the genotypes of the mice were verified (Fig. 3a, b). Western blot analysis confirmed that GCH1 expression and protein phosphorylation were decreased in *Gch1*-CKO mice (Fig. 3c, d). To determine whether *GCH1* KO affects radiation-induced lipid peroxidation, the concentration of MDA in lung tissues after 15 Gy irradiation was measured as previously reported^40^. After thoracic irradiation, ROS levels were higher in *Gch1*-CKO mice than in control mice (Fig. 3e). As shown in Fig. 3f, MDA levels were significantly increased in the lung tissues of *Gch1*-CKO mice compared with those of control mice following irradiation, indicating that GCH1 influences lipid peroxidation resulting from radiation-induced oxidation and/or amplification by autoxidation. Compared with those in control mice, the NO levels in the lung tissues of *Gch1*-CKO mice were decreased (Fig. 3g).

To further confirm the role of GCH1 in radiation-induced injury, conditional *Gch1* knock-in mice (*Gch1*^*lsl/lsl*^; *Sftpa1-Cre*^*+/−*^ mice) were generated (Fig. 3h–k). Compared with those in control mice, the radiation-induced ROS and MDA levels in the lung tissues of *Gch1*-CKI mice were significantly decreased (Fig. 3l, m). Consistently, NO levels in the lung tissues of *Gch1*-CKI mice were increased after 15 Gy irradiation (Fig. 3n). In summary, *GCH1* overexpression ameliorated radiation-induced acute lung injury in mice.

Finally, we established a mouse model of radiation-induced lung injury by unilateral pulmonary irradiation (Fig. 3o, p). After injection of Ad-EGFP via the tail vein, strong signals were observed in the liver and lungs, indicating that tail vein injection of the adenovirus could manipulate pulmonary gene expression (Supplementary Fig. 7a, b). To investigate the therapeutic effect of manipulating the GCH1/BH4 axis in acute radiation-induced lung injury, the mice were injected with Ad-GCH1, BH4 (1 mg/kg) or control agents via the tail vein after 20 Gy irradiation. Seven days after irradiation, the mice were sacrificed, and lung tissues were collected. Western blot analysis confirmed a marked increase in GCH1 expression in Ad-GCH1-injected mice (Fig. 3q). Exposure to 20 Gy of irradiation significantly increased ROS levels in the right lung, whereas infection with Ad-GCH1 but not Ad-NC reduced ROS levels (Fig. 3r). The results of BH4 injection were consistent with those of Ad-GCH1 (Fig. 3r). As shown in Fig. 3s, MDA levels were significantly lower in the right lung tissues of mice treated with Ad-GCH1 or BH4 than in the lung tissues of mice treated with Ad-NC or PBS following irradiation. NO levels in right lung tissues after 20 Gy irradiation were increased by *GCH1* overexpression or BH4 supplementation (Fig. 3t). In nonirradiated left lung tissues, ROS, MDA and NO levels were generally unchanged by Ad-GCH1 infection or BH4 supplementation (Supplementary Fig. 7c–e). Radiation exposure activated p-Smad2/3 (S423/425) and upregulated TGF-β1, whereas Ad-GCH1 administration or BH4 supplementation significantly decreased the levels of these fibrotic markers (Supplementary Fig. 7f, g). Taken together, these results indicate that the GCH1/BH4 axis is a therapeutic target for the treatment of radiation-induced acute lung injury.

### *GCH1* regulates radiation-induced pulmonary fibrosis

**Fig. 4:**
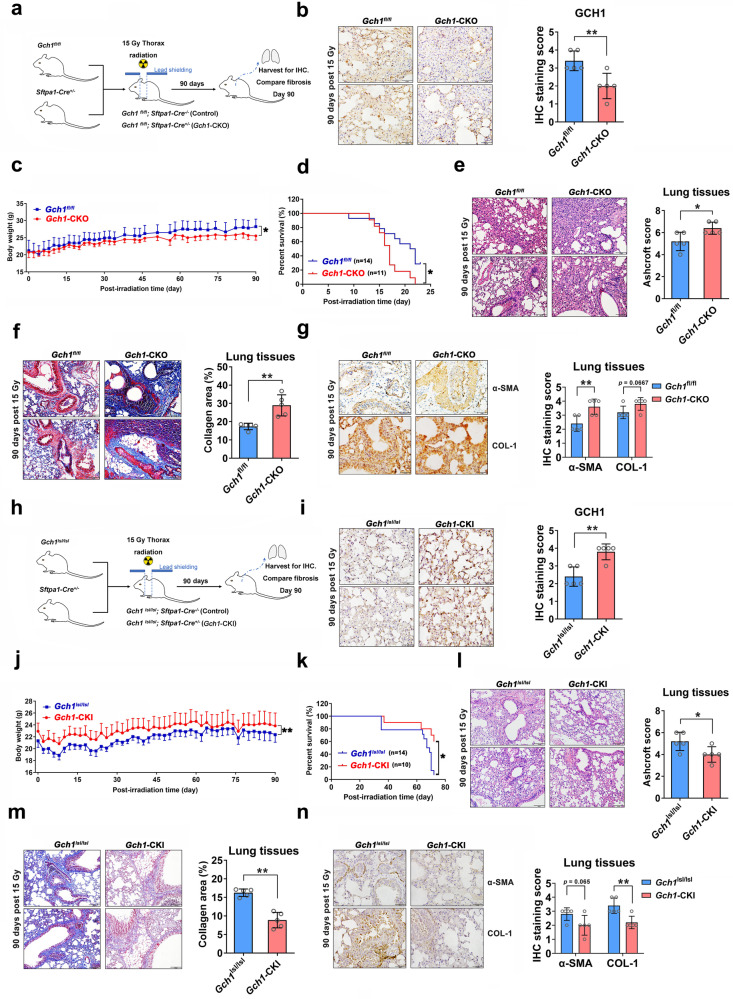
*GCH1* influences radiation-induced pulmonary fibrosis. **a** Experimental scheme for *Gch1*-CKO mouse breeding and treatment. **b** Immunostaining analysis of GCH1 expression in the lung tissues of mice. Scale bar = 50 μm. Quantitative analysis of GCH1-positive cells in the lung tissues of *Gch1*-CKO mice. **c** The weights of the mice in each group were monitored from Day 0 to Day 90. **d** Compared with control mice, GCH1 KO mice had shortened survival times (*n* = 10–14 for each group). **e** Representative H&E staining of mouse lungs. Scale bar = 100 μm. A Bar graph showing the Ashcroft scores of H&E-stained lung tissues from *Gch1*-CKO mice. **f** Representative Masson’s trichrome staining of mouse lungs. Scale bar = 100 μm. Quantification of the collagen deposition area in *Gch1*-CKO mouse lung tissues. **g** Lung tissues were immunostained for α-SMA and COL-1 and counterstained with hematoxylin. Scale bar = 50 μm. Quantitative analysis of α-SMA- and COL-1-positive cells in the lung tissues of *Gch1*-CKO mice. **h** Experimental scheme for the treatment of *Gch1*-CKI mice. **i** Immunostaining analysis of GCH1 expression in the lung tissues of mice. Scale bar = 50 μm. Quantitative analysis of GCH1-positive cells in the mung tissues of *Gch1*-CKI mice. **j** The weights of the mice in each group were monitored from Day 0 to Day 90. **k** Compared with control mice, GCH1 knock-in mice had prolonged survival times (*n* = 10–14 for each group). **l** Representative H&E staining of mouse lungs. Scale bar = 100 μm. A Bar graph showing the Ashcroft scores of H&E-stained lung tissues from *Gch1*-CKI mice. **m** Representative Masson’s trichrome staining of mouse lungs. Scale bar = 100 μm. Quantification of the collagen deposition area in *Gch1*-CKI mouse lung tissues. **n** The expression of α-SMA and COL-1 in lung tissues. Scale bar = 50 μm. Quantitative analysis of α-SMA- and COL-1-positive cells in the lung tissues of *Gch1*-CKO mice. *P < 0.05 and **P < 0.01 compared with the control group.

Since GCH1-mediated anabolism has been shown to regulate the fibrotic phenotype in vitro, we examined whether GCH1 affected radiation-induced pulmonary fibrosis in vivo. A mouse model of radiation-induced lung injury induced by 15 Gy of thoracic irradiation was established as described previously (Fig. 4a). GCH1 expression was decreased in the lung tissues of *Gch1*-CKO mice (Fig. 4b). On the 90th day postirradiation, a marked decrease in body weight was observed in *Gch1*-CKO mice (Fig. 4c). The lifespan of *Gch1*-CKO mice was significantly shorter than that of control mice (Fig. 4d). *GCH1* KO increased alveolar septal thickness and structural damage (Fig. 4e) and aggravated the deposition of collagen (Fig. 4f). The expression of α-SMA and COL-1 was increased in the lung tissues of *Gch1*-CKO mice, as determined by immunostaining (Fig. 4g).

A *Gch1*-CKI mouse model was also established (Fig. 4h). Compared with control mice, *Gch1*-CKI mice showed a marked increase in GCH1 expression, as confirmed by immunohistochemistry (Fig. 4i). Surprisingly, the *Gch1*-CKI mice had significantly heavier body weights and longer survival times than control mice following irradiation (Figs. 4j, k). Compared with control mice, *Gch1*-CKI mice exhibited fewer multifocal fibrotic lesions in their lung tissues on Day 90 after irradiation (Fig. 4l). *GCH1* knock-in also reduced the deposition of collagen (Fig. 4m) and the expression of α-SMA and COL-1 (Fig. 4n). Taken together, these results indicate that GCH1 plays an important role in radiation-induced lung fibrosis.

### BH4 metabolism attenuates radiation-induced pulmonary fibrosis in vivo

**Fig. 5:**
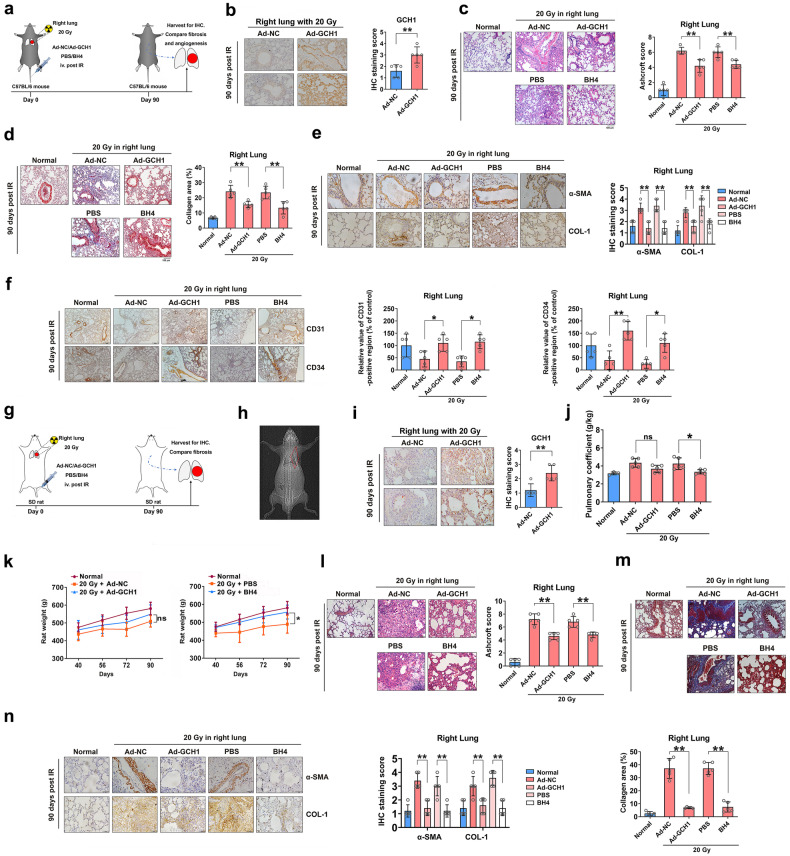
BH4 metabolism ameliorates radiation-induced pulmonary fibrosis in vivo. The right lung was not irradiated or was irradiated with a single dose of 20 Gy of X-ray irradiation, after which the mice were injected with Ad-NC or Ad-GCH1 and PBS or BH4 (five animals per group). **a** Experimental scheme showing the mouse treatments. **b** GCH1 levels in lung tissues in the Ad-NC and Ad-GCH1 group. Scale bar = 50 μm. **c** Representative H&E staining of mouse lungs in the various groups 90 days after irradiation. Scale bar = 100 μm. A Bar graph showing the Ashcroft scores of H&E-stained mouse lung tissues. **d** Representative Masson’s trichrome staining of mouse lungs. Scale bar = 100 μm. Quantification of the collagen deposition area in mouse lung tissues. **e** Lung tissues were immunostained for α-SMA and COL-1 and counterstained with hematoxylin. Scale bar = 50 μm. Quantitative analysis of α-SMA- and COL-1-positive cells in mouse lung tissues. **f** Microvessel density was visualized by CD31 and CD34 immunohistochemical staining in the right lung tissues of mice. The right lungs of the rats were not irradiated or were irradiated with a single dose of 20 Gy X-ray irradiation followed by in vivo injection of Ad-NC or Ad-GCH1 or PBS or BH4 (five animals per group). **g** Experimental scheme showing the treatments. **h** Representative CT image of the rats before radiation. **i** Immunostaining analysis of GCH1 expression in the lung tissues of rats. Scale bar = 50 μm. **j** The weights of the rats in each group were monitored from Day 40 to Day 90. **k** Pulmonary coefficients of each group. The pulmonary coefficient was calculated as the lung weight (g)/rat weight (kg). **l** Representative H&E staining of rat lung tissues in the various groups. Scale bar = 100 μm. A Bar graph showing the Ashcroft scores of H&E-stained sections of rat lung tissues. **m** Representative Masson’s trichrome staining of rat lungs. Scale bar = 100 μm. Quantification of collagen deposition area in rat lung tissues. **n** Lung tissues were immunostained for α-SMA and COL-1 and counterstained with hematoxylin. Scale bar = 50 μm. Quantitative analysis of α-SMA- and COL-1-positive cells in rat lung tissues. *P < 0.05 and **P < 0.01 compared with the Ad-NC-injected control group or PBS-injected control group.

To further validate the role of GCH1 in radiation-induced pulmonary fibrosis and identify therapeutic strategies, a mouse model of radiation-induced lung injury induced by unilateral pulmonary irradiation was established as previously described (Fig. 5a). After exposure to a dose of 20 Gy of X-rays, the mice were injected with Ad-GCH1, BH4 or the respective vehicle controls via the tail vein. Ninety days after irradiation, lung specimens were collected, and immunohistochemistry confirmed a marked increase in GCH1 in the Ad-GCH1-injected group (Fig. 5b). As expected, in the Ad-NC and PBS groups, the alveolar cavity in the right lung was filled with cellulose, while nonirradiated lung tissue did not exhibit this feature (Fig. 5c). GCH1/BH4 alleviated multifocal fibrotic lesions, resulting in fewer organized and smaller foci and reduced septal enlargement (Fig. 5c). In the left lung, there was no significant difference among the groups (Supplementary Fig. 8a). In the right lung, *GCH1* overexpression or BH4 supplementation reduced the deposition of collagen (Fig. 5d) and the expression of α-SMA and COL-1 (Fig. 5e). Moreover, the GCH1/BH4 axis facilitated angiogenesis in irradiated lung tissues, as evidenced by the increased expression of CD31 and CD34 (Fig. 5f). Comparatively, there were no changes in the nonirradiated left lung in the different groups (Supplementary Fig. 8b–d).

To further verify the role of the GCH1/BH4 axis in radiation-induced lung injury, we constructed a rat model of radiation-induced lung injury by unilateral pulmonary irradiation with 20 Gy of radiation (Fig. 5g, h). The GCH1/BH4 axis ameliorated radiation-induced lung fibrosis (Fig. 5i–n), but no effect was observed on unirradiated left lung tissues (Supplementary Fig. 8e–g).

### BH4 restores protein S-nitrosylation that was altered by radiation

**Fig. 6:**
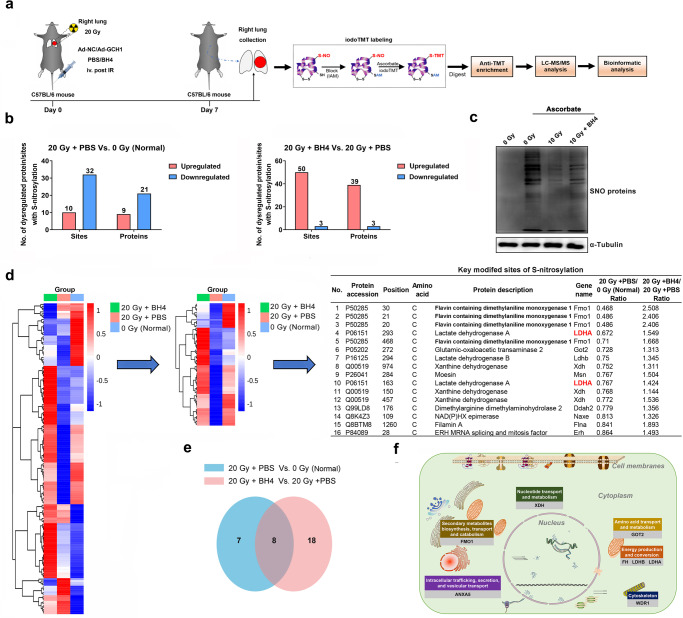
BH4 restores protein S-nitrosylation after irradiation. **a** Experimental design of the protein S-nitrosylation analysis. **b** (Left) Number of dysregulated S-nitrosylation sites and proteins with differential S-nitrosylation sites in mouse lungs exposed to 0 and 20 Gy of radiation. (Right) Number of dysregulated S-nitrosylation sites and proteins with differential S-nitrosylation sites between control (PBS)- and BH4-treated irradiated mouse lung tissues. **c** BH4 restored protein S-nitrosylation in HELF cells. Protein S-nitrosylation was determined by a biotin switch assay. The sample without ascorbate was representative of endogenous biotinylated protein levels. **d** IodoTMT-based quantitative protein S-nitrosylation analysis was used to identify S-nitrosylated proteins that were dysregulated in lung tissues. Key modified sites were identified. **e** Venn diagram showing proteins with S-nitrosylation in the two groups. **f** DAVID functional clustering and literature curation were performed to identify pathways targeted by protein S-nitrosylation.

Since NO homeostasis has been shown to regulate protein S-nitrosylation, which is an important posttranslational modification^41^, we hypothesized that BH4 could modulate protein S-nitrosylation following irradiation. The protein S-nitrosylation profile of mouse lung tissues was examined by iodoacetyl tandem mass tag (iodoTMT)-based protein quantification (Fig. 6a). A total of 194 S-nitrosylation sites were successfully identified in 122 proteins, of which 167 S-nitrosylation sites were identified in 107 proteins, and these sites contained quantitative information (Supplementary Table 1). Compared with those in the 0 Gy control group, 10 upregulated S-nitrosylation sites were found in 9 proteins and 32 downregulated S-nitrosylation sites were found in 21 proteins in the 20 Gy-irradiated group. Compared with those in the 20 Gy-irradiated group, the BH4-treated group had 50 upregulated S-nitrosylation sites in 39 proteins and 3 downregulated S-nitrosylation sites in 3 proteins (Fig. 6b). These results indicated that BH4 levels were associated with global protein S-nitrosylation. To confirm this finding, cellular protein S-nitrosylation was determined by a biotin switch assay. Total S-nitrosylation of cellular proteins was decreased in 10 Gy-irradiated HELF cells, whereas total S-nitrosylation was partially restored after BH4 supplementation following irradiation (Fig. 6c). We therefore targeted proteins in which S-nitrosylation was reduced after radiation and restored after BH4 supplementation. Key modified proteins, including flavin-containing dimethylaniline monooxygenase 1 (Fmo1), lactate dehydrogenase A (LDHA) and xanthine dehydrogenase (Xdh), were identified (Fig. 6d). The localization and functional classification of BH4-affected S-nitrosylated proteins are shown in Fig. 6e, f and Supplementary Fig. 9a, b. Taken together, these results revealed that BH4 influenced global protein S-nitrosylation in irradiated lungs.

### BH4 regulates ROS generation through LDHA S-nitrosylation

**Fig. 7:**
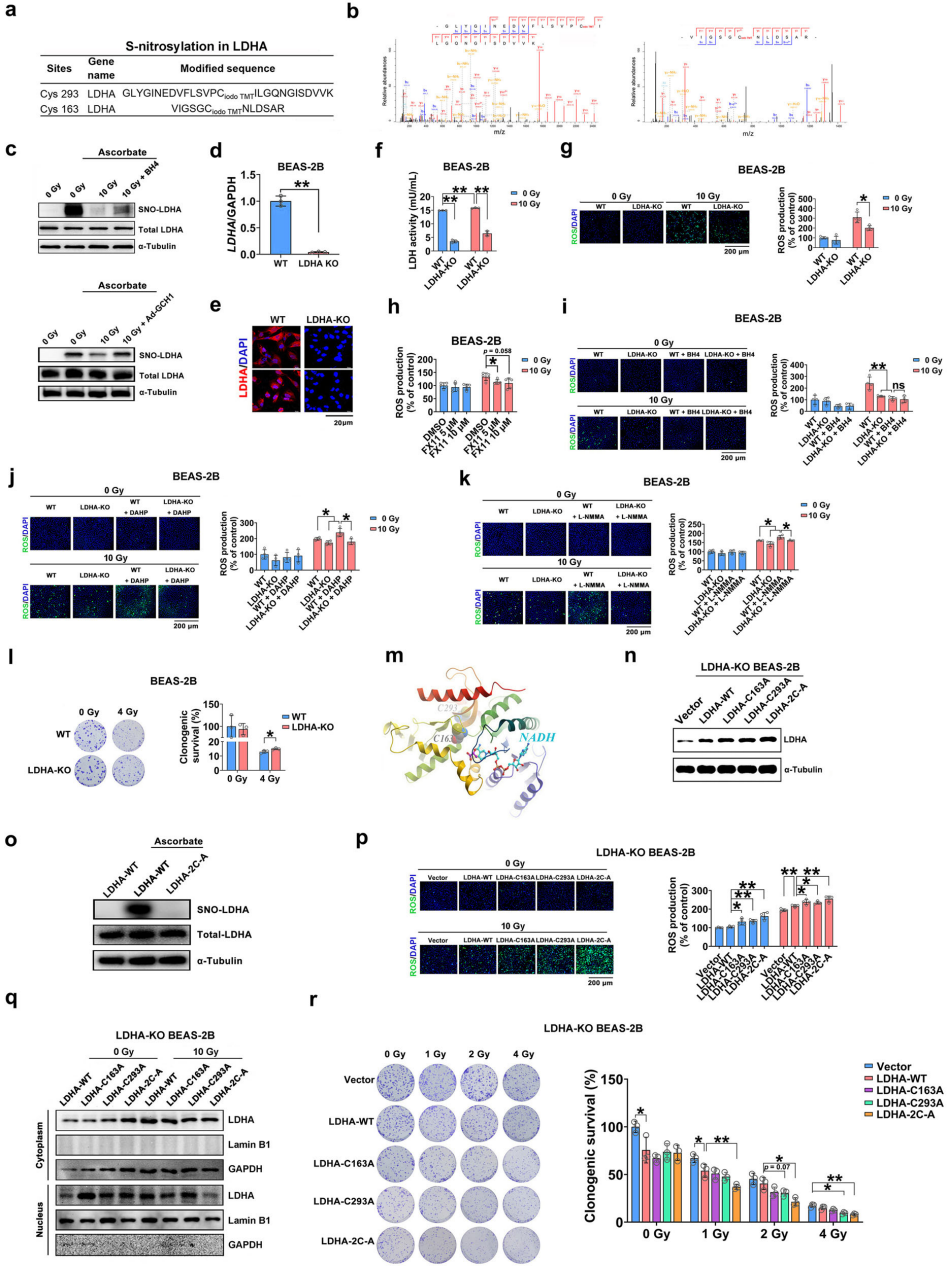
*LDHA* mediates ROS generation by BH4 through S-nitrosylation. **a**, **b** S-nitrosylation of LDHA (Cys163 and Cys293) was examined by LC–MS/MS. Sequence-informative fragmentation ions are summarized based on the peptide sequence and are annotated in blue (b-ions) and red (y-ions). **c** S-nitrosylated LDHA in HELF cells was analyzed by a biotin switch assay followed by Western blot analysis. The sample without ascorbate was representative of endogenous biotinylated protein levels. **d** The mRNA levels of LDHA in parental and LDHA-KO cells. **e** Immunofluorescence staining for LDHA (red) and DAPI (blue) in BEAS-2B cells. Scale bar = 20 μm. **f** LDH activity was examined in BEAS-2B cells. **g** ROS levels in BEAS-2B cells were determined using a fluorescence microscope or 96-well plate reader. Scale bar = 200 μm. **h** *LDHA*-KO BEAS-2B cells were incubated with FX11 followed by 10 Gy of X-ray irradiation. ROS levels in BEAS-2B cells were determined using a 96-well plate reader. **i** *LDHA* KO BEAS-2B cells were treated with BH4 followed by 10 Gy of X-ray irradiation. ROS levels in BEAS-2B cells were determined using a fluorescence microscope or 96-well plate reader. Scale bar = 200 μm. **j** *LDHA*-KO BEAS-2B cells were incubated with the GCH1 inhibitor DAHP (50 μM) and then exposed to 10 Gy of X-ray irradiation. ROS levels were determined using a fluorescence microscope or 96-well plate reader. **k** *LDHA*-KO BEAS-2B cells were incubated with the NOS inhibitor L-NMMA (100 μM) and then exposed to 10 Gy of X-ray irradiation. ROS levels were determined using a fluorescence microscope or 96-well plate reader. BEAS-2B cells were transfected with WT LDHA or the mutants followed by 10 Gy of X-ray irradiation. **l** The clonogenic survival of parental and *LDHA*-KO BEAS-2B cells was measured. **m** Crystal structure of LDHA bound to NADH and D-lactate (PDB entry 4JNK). The proteins are shown in a rainbow ribbon representation. NADH is shown as sticks with carbons colored cyan and d-lactate colored magenta. The residues Cys163 and Cys293 are highlighted with balls. The residue number was corrected for the first amino acid, which was missing in the X-ray structure. **n** Western blot analysis of LDHA expression in *LDHA*-KO BEAS-2B cells transfected with the indicated vectors. **o** *LDHA*-KO BEAS-2B cells were transfected with WT LDHA or mutant LDHA (with double mutations at Cys163 and Cys298) (2C-A). S-nitrosylated LDHA was analyzed by using a biotin switch assay followed by western blotting. The sample without ascorbate was representative of endogenous biotinylated protein levels. **p** ROS levels in BEAS-2B cells were measured in the presence of GSNO (200 μM) using a fluorescence microscope or a 96-well plate reader. **q** Cells were transfected with WT LDHA or the mutants followed by radiation. The nuclear and cytoplasmic levels of LDHA were examined by Western blot analysis. **r** The clonogenic survival of *LDHA*-KO BEAS-2B cells was measured. *P* < 0.05 and **P < 0.01 compared with the control group.

LDHA, which is a redox-related enzyme, is involved in ROS production through its interaction with NADH^42,43^. Thus, this protein and its S-nitrosylation were studied in greater detail. Cys293 and Cys163 of mouse LDHA were S-nitrosylated (Figs. 7a, b). To further validate the S-nitrosylation of LDHA in response to radiation and BH4 in vitro, a biotin switch assay^44^ was used to measure the levels of S-nitrosylation in proteins in HELF cells. We found that the S-nitrosylation of LDHA was reduced in irradiated HELF cells, and this effect was partially reversed in BH4-treated cells and Ad-GCH1-infected cells (Fig. 7c). This result was consistent with the results of the iodoTMT-based quantitative protein S-nitrosylation analysis of our mouse model.

To characterize the role of S-nitrosylation of LDHA in ROS production, we constructed *LDHA* knockout (KO) BEAS-2B cells by using CRISPR/Cas9 technology (Supplementary Fig. 10a, b). *LDHA* depletion was confirmed by qRT‒PCR and immunofluorescence analysis (Fig. 7d, e) and resulted in significantly decreased LDH activity (Fig. 7f). Intriguingly, *LDHA* depletion significantly inhibited radiation-induced ROS production (Fig. 7g), indicating that LDHA plays a role in radiogenic ROS generation. Consistent with these findings, LDHA inhibition by FX11 decreased radiogenic ROS levels (Fig. 7h). The addition of BH4 did not further reduce the levels of radiation-induced ROS in *LDHA*-KO cells, suggesting that LDHA was the downstream effector of radiogenic ROS generation (Fig. 7i). Consistent with these findings, the increase in ROS generation induced by pretreatment with the GCH1 inhibitor DAHP was abrogated by *LDHA* silencing after irradiation (Fig. 7j).

Our results showed that NOS activity was essential for the attenuation of radiogenic ROS and superoxide anion production by the GCH1/BH4 axis (Fig. 1r, s and Supplementary Fig. 3 and 4). We next explored the effects of LDHA and NOS on the regulation of ROS generation. *LDHA* KO reduced the increase in ROS via L-NMMA (Fig. 7k). Additionally, *LDHA* depletion did not affect the production of NO in the presence or absence of radiation (Supplementary Fig. 11a). These findings further suggest that LDHA is downstream of BH4 and NOS. *LDHA* depletion significantly enhanced cell survival after irradiation (Fig. 7l).

Since Cys293 and Cys163 of mouse LDHA were S-nitrosylated, an interaction model of LDHA with NADH was established (Fig. 7m), and Cys293 was approximately 17 Å from the nicotinamide moiety of NADH. In contrast, Cys163 was within 9 Å and shielded by the loop consisting of residues from Glu192 to Ser196 (Fig. 7m). Presumably, reorganization of the loop exposed Cys163 and allowed the covalent addition of the NO moiety to the sulfur atom. BEAS-2B cells were transfected with plasmids encoding WT LDHA or mutant LDHA (C163A or C293A or simultaneous mutation (2C-A)). Transfection of WT LDHA or the LDHA mutants into *LDHA*-KO BEAS-2B cells induced *LDHA* overexpression (Fig. 7n). A biotin switch assay showed that mutations at Cys163 and Cys298 abrogated the S-nitrosylation of LDHA (Fig. 7o). S-nitrosoglutathione (GSNO), which is a major endogenous NO donor, has been suggested to induce SNO protein formation^45^. LDHA enhanced radiation-induced ROS production in the presence of GSNO (Fig. 7p). Notably, cells expressing the LDHA mutants exhibited increased ROS production compared to WT LDHA-overexpressing cells with or without radiation exposure (Fig. 7p), indicating that the loss of S-nitrosylation in LDHA facilitated ROS production. LDH activity was lower in cells transfected with LDHA mutants than in WT LDHA-overexpressing cells exposed to radiation (Supplementary Fig. 11b). Since the nuclear translocation of LDHA has been shown to activate antioxidant responses^42^, *LDHA*-KO BEAS-2B cells were transfected with WT LDHA or LDHA mutants and then subjected to a nuclear isolation assay. We found that LDHA translocated from the cytoplasm to the nucleus after irradiation (Fig. 7q), whereas the double mutation (C163A/C293A) weakened LDHA nuclear translocation (Fig. 7q). Finally, cells in which LDHA S-nitrosylation was removed had lower survival than LDHA-WT cells (Fig. 7r). These results indicated that LDHA S-nitrosylation could weaken the ability to generate ROS and thus enhance cell survival.

Previous studies suggested that LDH-NADH complexes could generate ROS through the chain oxidation of NADH initiated and propagated by superoxide (44). The increase in NADH oxidation is inhibited by superoxide dismutase (SOD)^46^. To determine whether this mechanism is independent of LDHA S-nitrosylation in the ROS cascade, cells were treated with BH4 and/or SOD1 plasmids, after which ROS production was measured in the presence or absence of radiation. The results showed that ROS levels in irradiated lung cells were decreased after BH4 administration or SOD1 overexpression. When BH4 administration was combined with SOD1 overexpression, radiation-induced ROS production was further reduced (Supplementary Fig. 11c, d). Thus, NADH oxidation and LDHA S-nitrosylation may contribute to the generation of ROS.

## Discussion

Recent studies have suggested that cellular redox metabolism is modulated in response to oxidative stress and plays a key role in exacerbating toxicity^3,4^. The accumulation of ROS during redox metabolism leads to biomolecular damage and even cell death. Herein, we demonstrated that BH4 was a redox-sensitive cofactor and that its biosynthesis as suppressed by ionizing radiation, which amplified ROS production. These findings are important for radiotherapy and represent one mechanism by which cells sense and amplify oxidative events, such as metabolic ROS generation. However, the mechanism by which irradiation decreased GCH1 expression remains unclear. The autophagy and ubiquitin‒proteasome systems are two main pathways associated with protein degradation^47^. Previous studies have shown that oxidative stress, including irradiation, stimulates ubiquitin–proteasomal protein degradation^48–50^. Jing et al.^51^ reported that the decrease in GCH1 expression in hyperoxia is partly due to increased degradation by the ubiquitin‒proteasome system, which is consistent with our findings. Genetic manipulation of BH4 metabolism by altering *GCH1* or direct supplementation with BH4 inhibited radiation-induced ROS production and attenuated lung injury in the acute stages but during fibrosis. These findings indicate a novel role of the GCH1/BH4 axis in the response to radiation. Since there is no effective approach for preventing radiation-induced lung injury in clinical practice^34,52–55^, the GCH1/BH4 axis could serve as a novel therapeutic target in the management of radiation-induced lung injury. GCH1 activity is regulated by phosphorylation, and phosphorylation is modulated by stimuli^30,56^. Notably, GCH1 phosphorylation at S81 was important for BH4 production^30^. S81 in GCH1 was replaced with an aspartate to mimic phosphorylation (S81D), and irradiated lung fibroblasts harboring this S81D mutant produced less radiogenic ROS and more NO than WT cells. Thus, GCH1 S81D is advantageous for the treatment of oxidative stress-induced injury. Due to the properties of endogenous BH4, further studies are needed to explore delivery approaches, including extending its stability to overcome radiosensitivity for clinical applications. It has been reported that vitamin C and folic acid can enhance the binding of BH4 to NOS, thereby increasing the level of intracellular BH4^57,58^. Improving the bioavailability of BH4 with liposomes has been reported to reverse the loss of myocardial NOS activity and BH4 levels after ischemia–reperfusion injury^59,60^. Targeted delivery of BH4 nanocarriers is also used as a prophylactic treatment for atherosclerosis^61^. Thus, delivery systems such as liposomes and other nanoparticles are promising approaches for enhancing BH4 stability and may be valuable in clinical applications to prevent radiation-induced lung injury.

In the present study, we showed that the role of BH4 in attenuating radiation-induced ROS and superoxide anion production depended on NOS. NOS possesses the unique ability to be “uncoupled” and produce superoxide anions instead of NO. The GCH1/BH4 axis modulates radiogenic ROS by inhibiting NOS uncoupling. eNOS activation depends on the formation of dimers^62^. In our study, neither GCH1 overexpression by an adenovirus nor BH4 addition increased eNOS dimer levels in HELF cells (data not shown). We hypothesize that the disruption of BH4 by radiation is not sufficient to affect eNOS dimerization.

NO can regulate cell signaling and fuel protein S-nitrosylation, which is an important posttranslational modification^19,20^. Dysregulated S-nitrosylation plays an increasingly important role in many human diseases^63,64^. We found that oxidative stress impairs the S-nitrosylation of multiple proteins, and BH4 restored the decrease in protein S-nitrosylation. Our results reveal a critical link between the oxidative response and protein S-nitrosylation and support the novel concept that oxidative stress alters protein S-nitrosylation through the GCH1/BH4 axis. LDHA, which can produce ROS^42,43^ via BH4-regulated protein S-nitrosylation, was studied in greater detail. LDHA has five cysteine residues, of which Cys163 and Cys293 were shown to undergo the loss of S-nitrosylation in response to irradiation. S-nitrosylation at Cys163 may affect the interaction with NADH and attenuate ROS production (Fig. 7m). Furthermore, mutation of the S-nitrosylation sites (Cys163 and/or Cys293) in LDHA enhanced ROS production and reduced LDH activity. Previous studies by other groups and our group indicate that ROS production is a result of NOS uncoupling^13,14,65^. Notably, in the present study, LDHA-mediated radiation-induced ROS production, as evidenced by the low ROS levels in *LDHA*-KO cells exposed to radiation (Fig. 7g). Thus, ROS amplification by radiation is not directly mediated by the BH4/NO axis but by the downstream effector LDHA through S-nitrosylation (Fig. 7i–k). Notably, NADH oxidation and LDHA S-nitrosylation can contribute to the generation of ROS (Supplementary Fig. 11c, d). These findings illustrate a novel mechanism by which the GCH1/BH4/LDHA axis drives ROS production. Since *LDHA* overexpression is related to cancer cell growth, poor prognosis and drug resistance, the inhibition of LDHA is an area of active research in the field of radiation oncology^66^. Additionally, the inhibition of LDHA can prevent and treat pulmonary fibrosis^67,68^. LDHA is involved in histone lactylation and regulates gene expression^69^. Thus, future studies on the role of LDHA-mediated protein lactylation in preventing radiosensitivity and the mechanism are warranted.

**Fig. 8:**
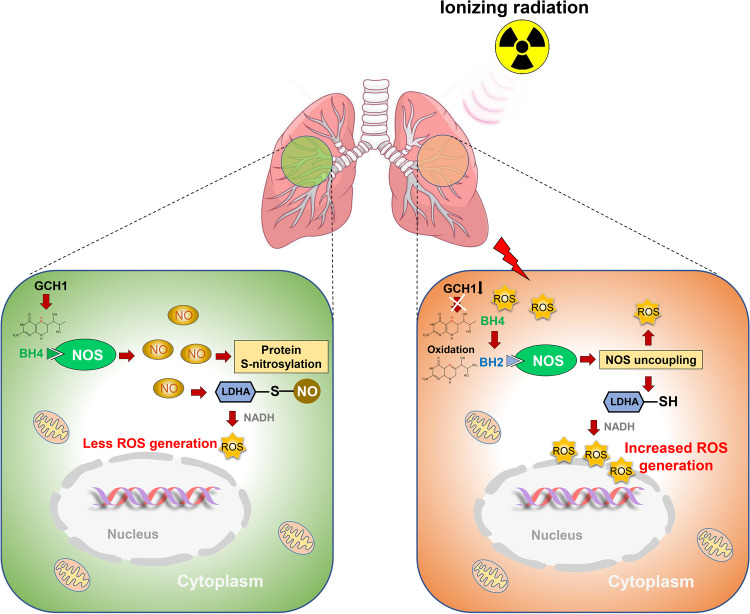
Schematic representation of GCH1-mediated BH4 metabolism during ROS generation. S-nitrosylation of LDHA-mediated by BH4 metabolism results in decreased ROS production. Ionizing radiation disrupts BH4, which causes NOS uncoupling, interrupts LDHA S-nitrosylation, and increases ROS production.

In summary, we demonstrated that radiation disrupts BH4 availability, which interrupts LDHA S-nitrosylation, leading to ROS amplification (Fig. 8). These findings reveal a novel, critical role of GCH1/BH4 axis-mediated LDHA S-nitrosylation in ROS generation and cellular radiosensitivity, which provides novel strategies for ameliorating radiation-induced lung injury.

## Supplementary information

Supplementary file

Supplementary Table 1

## Data Availability

The data that support the findings of this study are available from the corresponding author upon reasonable request.
