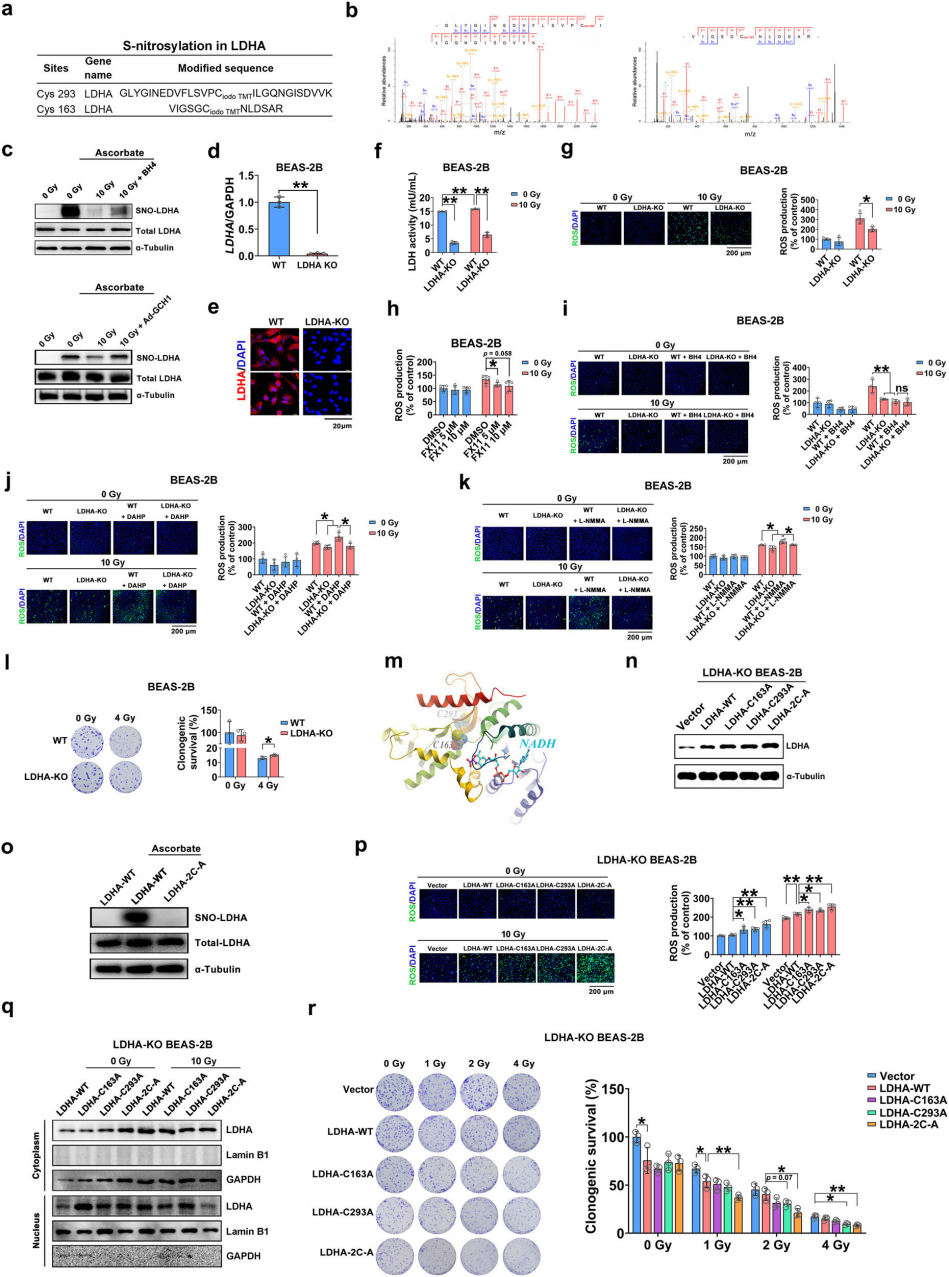# Author Correction: Tetrahydrobiopterin metabolism attenuates ROS generation and radiosensitivity through LDHA S-nitrosylation: novel insight into radiogenic lung injury

**DOI:** 10.1038/s12276-025-01484-3

**Published:** 2025-07-14

**Authors:** Yang Feng, Yahui Feng, Liming Gu, Wei Mo, Xi Wang, Bin Song, Min Hong, Fenghao Geng, Pei Huang, Hongying Yang, Wei Zhu, Yang Jiao, Qi Zhang, Wei-Qun Ding, Jianping Cao, Shuyu Zhang

**Affiliations:** 1 State Key Laboratory of Radiation Medicine and Protection, School of Radiation Medicine and Protection, Medical College of Soochow University, 215123 Suzhou, China; 2 Department of Oncology, Wuxi No.2 People’s Hospital, Jiangnan University Medical Center, 214002 Wuxi, China; 3 Laboratory of Radiation Medicine, Second Affiliated Hospital of Chengdu Medical College, China National Nuclear Corporation 416 Hospital, 610051 Chengdu, China; 4 West China Second University Hospital, Sichuan University, 610041 Chengdu, China; 5 Department of Pathology, Stephenson Cancer Centre, College of Medicine, University of Oklahoma Health Sciences Center, Oklahoma City, OK 73104 USA; 6 Laboratory of Radiation Medicine, West China School of Basic Medical Sciences & Forensic Medicine, Sichuan University, 610041 Chengdu, China; 7 NHC Key Laboratory of Nuclear Technology Medical Transformation (Mianyang Central Hospital), 621099 Mianyang, China

**Keywords:** Post-translational modifications, Molecular biology

Correction to: *Experimental & Molecular Medicine* 10.1038/s12276-024-01208-z, published online 01 May 2024

After online publication of this article, the authors noticed an error in the Fig. 7k control group (0 Gy with LDHA-KO + L-NMMA), caused by accidentally overwriting the original merge image file during assembly.

The figure should have appeared as shown below.