# Supplementary material for: Tetrahydrobiopterin metabolism attenuates ROS generation and radiosensitivity through LDHA S-nitrosylation: novel insight into radiogenic lung injury
Source: Exp Mol Med. 2024 May 1;56(5):1107–22. doi: 10.1038/s12276-024-01208-z (PMC11148139; doi:10.1038/s12276-024-01208-z)
Supplement: Supplementary file 1 — Supplementary file [file 12276_2024_1208_MOESM1_ESM.pdf]

Supplementary file list

1. Supplementary Materials and Methods

2. Supplementary Figure and Figure legends

3. Supplementary Table 1

Supplementary materials and methods

Reagents and materials

BH4 was obtained from the Cayman Chemical Company (Ann Arbor, MI). Recombinant human TGF-β1 was purchased from PeproTech (Rocky Hill, NJ). FX11, DAHP and MG-132 were obtained from MedChemExpress (Monmouth Junction, NJ). Ferrostatin-1 was purchased from Sigma–Aldrich (St. Louis, MO). The control adenovirus (Ad-NC) and adenoviruses encoding either wild-type (WT) GCH1 (Ad-GCH1) or GCH1 in which S81 was replaced with an aspartate to mimic phosphorylation (Ad-GCH1-S81D) or an alanine to block phosphorylation (Ad-GCH1-S81A) were purchased from Vigene Biosciences (Jinan, China). siRNA targeting *GCH1* and S-nitrosoglutathione (GSNO) was obtained from Santa Cruz Biotechnology (Santa Cruz, CA). Enzyme-linked immunosorbent assay (ELISA) kits for BH4 and BH2 were purchased from Mmbiology (Yancheng, China). L-NMMA and L-NAME were purchased from GlpBio (Montclair, CA). Antibodies against GAPDH and α-Tubulin and HRP-conjugated anti-mouse and anti-rabbit IgG were purchased from Beyotime (Nantong, China). Biotin switch assay kit (#ab236207), LDH assay (#ab102526) and antibodies against GCH1 (#ab61858), TGF-β1 (#ab64715), phospho-Smad2

(#ab53100), phospho-Smad3 (#ab52903), COL-1 (#ab34710), LDHA (#ab135396) and SOD1 (#ab51254) were purchased from Abcam (Cambridge, MA). Antibodies against Smad2 (#12570-1-AP), PTPS (#12150-1-AP), SR (#16822-1-AP) and GFRP (#118809-1-AP) were purchased from Proteintech (Chicago, IL). Antibodies against α-SMA (#YT5121), CD31 (#YT0752) and CD34 (#YT0757) were purchased from Immunoway (Newark, DE). Antibodies against eNOS (#CY3412), iNOS (#CY5993) and nNOS (#CY5342) were purchased from Abways (Shanghai, China). The anti-GCH1 phospho-Ser81 antibody was produced by Chengdu Zen Bioscience (Chengdu, China) in rabbits as follows. New Zealand white rabbits (Chengdu Dossy Experimental Animals Co. Ltd. China) were inoculated with a keyhole limpet haemocyanin-conjugated polypeptide (SSILS(pS)LGENPQRC-KLH, Chinese Peptide Company) spanning the Ser81 phosphorylation site in the human GCH1 protein. Antibody titers were measured by ELISA. After eight weeks, blood serum of the rabbits was collected and then purified by affinity chromatography using Sepharose-immobilized peptide to produce the anti-GCH1 phospho-Ser81 antibody. The human LDHA (GenBank accession No. NM_005566.3) coding region and human SOD1 (GenBank accession No. NM_000454.5) coding region were amplified by PCR using primer pairs specific for LDHA and SOD1, respectively. The amplified fragment was inserted into a pcDNA 3.1 vector. The plasmid was then sequenced for confirmation. Vectors of LDHA mutants (one point mutation C163A or C293A and double mutation C163A/C293A) with the C-terminal Flag tag were constructed by PPL Biotech (Nanjing, China).

NOS activity assay

NOS activity was measured with an NOS activity assay kit (Beyotime, Nantong, China). Briefly, HELF and BEAS-2B cells were plated onto 96-well plates. After radiation, cells were treated with 100 μL NOS detection buffer and 100 μL NOS detection reaction solution and incubated for 1 h. NOS activity was detected at 495 nm by a 96-well plate reader.

Superoxide production assay

Superoxide production was detected using dihydroethidium (DHE, Shanghai Bioscience, Shanghai, China). Briefly, HELF and BEAS-2B cells were washed with PBS and then incubated with 10 μM DHE for 30 min in DMEM. Fluorescence was measured using a fluorescence microscope (Leica IX73, Hessian, Germany). For quantification, superoxide production in cells was detected using a 96-well plate reader (Ex/Em = 480-535 nm/590-610 nm).

Clonogenic survival assay

HELF and BEAS-2B cells were plated onto 6-well plates at 200 cells/well. The clonogenic survival assay of cells was performed as reported previously [1]. Then, the cells were infected with Ad-GCH1 or treated with BH4 followed by 0 or 2 Gy of X-ray radiation. Following 10-14 days of incubation, the cells were fixed in methanol, followed by crystal violet staining. The number of colonies per well was counted, and the survival fractions were calculated as the relative plating efficiencies of the treated cells compared with that of the mock-irradiated (0 Gy) cells. Colonies consisting of 50 or more cells were counted as a clone.

Luciferase assays

To measure the activity of the TGF-β signalling pathway, a TGF-β downstream effector

Smad2/3-responsive luciferase reporter was used for the luciferase assay, as reported previously [2], reflecting the transcriptional activity of Smad2/3. Cells were transfected with luciferase reporter and internal control pRL-TK (Promega, Madison, WI) using ExFect Transfection Reagent (Vazyme, Nanjing, China). Luciferase activity was detected using the Dual-Luciferase Reporter Assay System (Promega). Promoter activities were expressed as the ratio of *Firefly* luciferase and *Renilla* luciferase activities.

Western blotting analysis

The origin of antibodies for Western blotting analysis has been described in the Reagents and materials section. Western blotting was used to detect changes in GCH1, phosphorylated GCH1 (P-Ser 81 GCH1), PTPS, SR, TGF-β1, phosphorylated Smad2 (p-Smad2), phosphorylated Smad3 (p-Smad3), α-SMA, Vimentin, E-cadherin, LDHA, GAPDH and α-Tubulin levels in lung tissue and HELF or BEAS-2B cells. In brief, cells and lung tissue were lysed in RIPA buffer (with protease inhibitor and phosphatase inhibitor). After centrifugation at 4°C for 15 min (12,000 x g), the supernatant was collected for Western blotting. Protein was fractionated by 10% SDS–PAGE, transferred to polyvinylidene difluoride membranes (EMD Millipore, Bedford, MA) and probed overnight at 4°C with primary antibodies: anti-GCH1 (1: 1000), anti-p-Ser 81 GCH1 (1: 1000), anti-PTPS (1: 1000), anti-SR (1: 1000), anti-TGF-β1 (1: 1000), anti-p-Smad2 (1: 500), anti-p-Smad3 (1: 2000), anti-LDHA (1:1000), anti-SOD1 (1:1000), anti-GAPDH (1:1000) and anti-α-Tubulin (1: 1000). Membranes were washed with PBST four times and incubated with HRP-conjugated secondary antibodies (goat anti-mouse or goat anti-rabbit, 1: 3000; Beyotime, Nantong, China) at room temperature for 1 h. Proteins were detected by

enhanced chemiluminescence (ECL; Beyotime).

Immunofluorescence assay

The cells were fixed with 4% formaldehyde and blocked with 3% BSA/PBS for 1 h at room temperature. Antibodies against Smad2 (1:200) were incubated with cells overnight at 4°C, followed by Cy3-labelled goat-anti-rabbit secondary antibodies (1:1000) for 1 h at room temperature. DAPI was used to stain the cell nuclei, and images were captured using confocal scanning laser microscopy (Olympus, Tokyo, Japan).

Enzyme-linked immunosorbent assay (ELISA)

After BH4 aqueous solution or rat lung tissues were exposed to 10 or 20 Gy X-ray, they were measured using a kit based on ELISA competition to assay BH4 level according to the manufacturer's instructions. The optical absorbance of the samples was measured at 450 nm.

Cell viability assay

Cell viability was determined by the CCK-8 assay (Cell Counting Kit-8; Beyotime, Nantong). Briefly, HELF cells were seeded into 96-well plates in triplicate and incubated at 37°C for 24 h. Then, the cells were infected with adenovirus for 24 h. The cells were exposed to 10 and 20 Gy of ionizing radiation. Twenty-four, 48 and 72 h later, 10 μL CCK-8 solution was added to the cells and then the cells were incubated at 37°C for 1 h, the absorbance at 492 nm was measured with a s microplate reader.

Quantitative real-time polymerase chain reaction (qRT–PCR)

Total RNA was extracted from BEAS-2B cells using TRIzol reagent (Invitrogen). Reverse transcription was performed using total RNA reverse transcribed using the PrimeScript RT

kit (Takara) according to the manufacturer's instructions. qRT–PCR was performed using the SYBR Green Master Mix Kit (Takara) on the ABI ViiA 7 Real-Time PCR system (Applied Biosystems, Foster City, CA, USA). The $2^{-\Delta\Delta}$CT method with GAPDH was used to normalize the fold change in expression to the endogenous control. The primer sequences used were as follows: *LDHA*, forwards ATGGCAACTCTAAAGGATCAGCTGATT and reverse TGATGGCACAGGCCATGCCAA; *GCH1* forwards ACGAGCTGAACCTCCCTAAC and reverse GAACCAAGTGATGCTCACACA; *GAPDH*, forwards GTCTCCTCTGACTTCAACAGCG and reverse ACCACCCTGTTGCTGTAGCCAA.

CRISPR–Cas9 gene editing

The Cas9 plasmid and sgRNA plasmid were constructed and transfected into BEAS-2B cells. The positive cells were screened by puromycin. After identification and amplification, a monoclonal cell line with LDHA gene knockout was obtained. The targeting sequences were as follows:

LDHA sgRNA-1: 5'- GTCCAATATGGCAACTCTAAAGG-3'

LDHA sgRNA-2: 5'- ACAACTGTAATCTTATTCTGGGG -3'

LDHA sgRNA-3: 5'- TACCTTCATTAAGATACTGATGG -3'

LDHA sgRNA-4: 5'- ACATTCATTCCACTCCATACAGG -3'

LDHA sgRNA-5: 5'- CTGATAAAGATAAGGAACAGTGG -3'

LDHA sgRNA-6: 5'- TTCACAAGCAGGTGGTTGAGAGG -3'

Transgenic mice

Gch1-floxed mice were generated by GemPharmatech (Nanjing, China) using the

CRISPR-CAS9 system. Cas9 mRNA, single guided RNA (sgRNA) and donor were coinjected into the fertilized egg. SgRNAs guided Cas9 endonuclease cleavage in the GCH1 intron, resulting in the insertion of LoxP sites into the GCH1 intron between exon 1 and exon 2 and between exon 2 and 3, respectively, through homologous recombination, and exon 2 was deleted. To obtain lung-specific knockout mice, floxed *Gch1* mice were hybridized with *Sftpa1*-cre transgenic mice. *Sftpa1*-cre vector was inserted randomly into mouse chromosomes, not *in situ* in Chromosome 14.

For the lung-specific overexpression of *Gch1*, the transgenic vector CAG-LSL-*Gch1*-IRES-EGFP-poly(A) was constructed. The homologous arms of the H11 locus were amplified by PCR using C57BL/6 mouse genomic DNA as the template. The CAG-LSL-*Gch1*-IRES-EGFP-poly(A) was inserted at the H11 site. A stop cassette upstream of *Gch1* was removed by Cre, which triggered the expression of Gch1.

Rats and irradiation

Protocols for experiments involving animals were approved by the Animal Experimentation Ethics Committee at Soochow University (Suzhou, China). Male SD rats (250-300 g) were purchased from Shanghai SLAC Laboratory Animal Co., Ltd. (Shanghai, China). A model of radiation-induced lung injury in rats by unilateral pulmonary irradiation was established as reported previously [3]. The right lung of each rat was irradiated with 6 MV X-rays (Varian 23EX linear accelerator, Palo Alto CA) at a single dose of 0, 10 or 20 Gy (n = 5), and the dose rate was 5 Gy/min. The left chest and other parts of the body were protected with 3 mm of lead. Correct positioning of the fields was controlled for each rat using a therapy simulator (Huestis Cascade Simulator, Bristol, RI) based on previous reports [3].

For the treatments, rats were divided randomly into five groups: 1) noninstrumented control group (n = 3); 2) 20 Gy-irradiated group injected with Ad-NC (n = 5); 3) 20 Gy-irradiated group injected with Ad-GCH1 (n = 5); 4) 20 Gy-irradiated group with administration of PBS (n = 5); and 5) 20 Gy-irradiated group with administration of BH4 (n = 5). After irradiation, rats in the irradiation groups separately received tail intravenous injections of 400 μL of Ad-NC (1.0×10^{12} vp/mL), Ad-GCH1 (1.0×10^{12} vp/mL), PBS and BH4 (1 mg/kg) immediately. During the experiment, the body weight of each rat was recorded. The rats were sacrificed, and samples were obtained on the 90th day after irradiation.

Measurement of malondialdehyde (MDA)

Total proteins were extracted from mouse lung tissues using RIPA buffer containing phenylmethanesulfonyl fluoride (PMSF). Then, lysed tissues were centrifuged, and the supernatant was subjected to the measurement of MDA levels using the MDA detection kit (JianCheng Biological Institution, Nanjing, China) and the protein contents by a BCA assay kit (Beyotime, Nantong, China). MDA levels were then normalized to milligrams of protein.

Immunohistochemistry (IHC)

Lung tissues were fixed in 10% neutral-buffered formalin and embedded in paraffin. Three-micrometre paraffin sections were deparaffinized and heat treated with citrate buffer (pH 6.0) for 7 min following an epitope retrieval protocol. Three-micrometre paraffin sections were incubated with antibodies against GCH1 (1: 1000), α-SMA (1: 100), COL-1 (1: 100), CD31 (1: 100) and CD34 (1: 100) at 4°C overnight followed by incubation with biotinylated secondary antibody (ZSGB-Bio, Beijing). IHC staining was visualized with substrate solution containing diaminobenzidine (DAB) and hydrogen peroxide. The counterstaining

was performed with haematoxylin. The sections were observed using a microscope (Leica IX73, Hessian, Germany).The criteria for scoring the stained sections were as follows: (1) negative, <10% of the whole tissue section stained positive or the section was almost not positive staining; (2) weakly positive, 10-25% of the whole tissue section stained positive or the section was stained light yellow; (3) moderately positive, 25-75% of the whole tissue section stained positive or the section was stained yellow; (4) strongly positive, 75% of the tissue section stained positive or the section was stained brown.

Protein extraction

Lung tissues from mice were snap-frozen in liquid nitrogen immediately after resection and stored at -80°C until use. The sample was ground by liquid nitrogen into cell powder. After that, four volumes of lysis buffer (0.5% SDS, 1% protease inhibitor and 50 mM IAM) were added to the cell powder, followed by sonication three times on ice using a high-intensity ultrasonic processor (Scientz). The remaining debris was removed by centrifugation at 12,000 x g at 4°C for 10 min. Finally, the supernatant was collected, and the protein concentration was determined with a BCA kit according to the manufacturer’s instructions.

Trypsin digestion

For digestion, the protein solution was reduced with 5 mM dithiothreitol for 30 min at 56°C and alkylated with 11 mM iodoacetamide for 15 min at room temperature in darkness. The protein sample was then diluted by adding 100 mM TEAB to urea concentrations less than 2M. Finally, trypsin was added at a 1:50 trypsin-to-protein mass ratio for the first digestion overnight and a 1:100 trypsin-to-protein mass ratio for a second 4 h digestion. The peptides digested by trypsin were desalted and freeze-dried in vacuo.

Iodoacetyl tandem mass tag (iodoTMT) labelling for protein S-nitrosylation profiling

Equal amounts of protein were taken from each sample, the volume was adjusted to the same with lysis buffer, 1 volume of precooled acetone was added, and then 4 volumes of precooled acetone were added after vortex mixing and precipitated for 2 h at -20°C. After centrifugation at 4500 x g for 5 min, the supernatant was discarded, and the precipitate was washed 3 times with precooled acetone. Finally, the protein precipitation was redissolved in buffer (50 mM HEPES pH 8.0, 1 mM EDTA, 0.1% SDS). The proteins were labelled according to the instructions of the iodoTMT kit. Briefly, after thawing, the labelled reagent was dissolved in methanol and mixed with the protein vortex. Then, a final concentration of 10 mM sodium ascorbate was added and incubated in darkness at 37°C for 1 h. After the labelled reaction, a final concentration of 20 mM DTT was added and incubated at 37°C in the dark for 15 min to terminate the reaction. The labelled protein was precipitated with 6x precooled acetone to remove excess reagents.

Affinity enrichment of protein S-nitrosylation

Tryptic peptides dissolved in NETN buffer (100 mM NaCl, 1 mM EDTA, 50 mM Tris-HCl, 0.5% NP-40, pH 8.0) were incubated with pre-washed anti-TMT antibody beads (Prod#90076, Thermo) at 4°C overnight with gentle shaking. Then, the beads were washed four times with NETN buffer and twice with H2O. The bound peptides were eluted from the beads with 0.1% trifluoroacetic acid. Finally, the eluted fractions were combined and vacuum-dried. For LC–MS/MS analysis, the resulting peptides were desalted with C18 ZipTips (Millipore) according to the manufacturer’s instructions.

Quantitative proteomic analysis by liquid chromatography tandem mass

spectrometry (LC‑MS/MS)

The tryptic peptides were dissolved in 0.1% formic acid (solvent A) and directly loaded onto a homemade reversed-phase analytical column (15-cm length, 75 μm i.d.). The gradient comprised an increase from 8% to 25% solvent B (0.1% formic acid in 90% acetonitrile) over 40 min, 23% to 35% in 12 min and climbing to 80% in 4 min then holding at 80% for the last 4 min, all at a constant flow rate of 500 nL/min on an EASY-nLC 1000 UPLC system.

The peptides were subjected to an NSI source followed by tandem mass spectrometry (MS/MS) in Q ExactiveTM Plus (Thermo) coupled online to the UPLC. The electrospray voltage applied was 2.0 kV. The m/z scan range was 350 to 1800 for the full scan, and intact peptides were detected in the Orbitrap at a resolution of 70000. Peptides were then selected for MS/MS using an NCE setting of 28, and the fragments were detected in the Orbitrap at a resolution of 17500. A data-dependent procedure alternated between one MS scan followed by 20 MS/MS scans with 15.0 s dynamic exclusion. Automatic gain control (AGC) was set at 5E4. The fixed first mass was set as 100 m/z.

Database search

The resulting MS/MS data were processed using the MaxQuant search engine (v.1.5.2.8). Tandem mass spectra were searched against the SwissProt Mus musculus database (20274 sequences) concatenated with the reverse decoy database. Trypsin/P was specified as the cleavage enzyme allowing up to 4 missing cleavages. The mass tolerance for precursor ions was set as 20 ppm in the first search and 5 ppm in the main search, and the mass tolerance for fragment ions was set as 0.02 Da. Carbamidomethyl on Cys was specified as a fixed modification, and methionine modification and oxidation on Met were

specified as variable modifications. The FDR was adjusted to < 1%, and the minimum score for modified peptides was set to > 40.

References

1. Xie, L.-W., Cai, S., Zhao, T.-S., Li, M., and Tian, Y. Green tea derivative (-)-epigallocatechin-3-gallate (EGCG) confers protection against ionizing radiation-induced intestinal epithelial cell death both in vitro and in vivo. *Free radical biology & medicine* **161**, 175-186 (2020).

2. Cao, J., Zhu, W., Yu, D., Pan, L., Zhong, L., Xiao, Y., Gao, Y., Jiao, Y., Zhang, Q., Ji, J., Yang, H., Zhang, S., and Cao, J. The Involvement of SDF-1α/CXCR4 Axis in Radiation-Induced Acute Injury and Fibrosis of Skin. *Radiation research* **192**, 410-421 (2019).

3. Xie, L., Zhou, J., Zhang, S., Chen, Q., Lai, R., Ding, W., Song, C., Meng, X., and Wu, J. Integrating microRNA and mRNA expression profiles in response to radiation-induced injury in rat lung. *Radiation oncology (London, England)* **9**, 111 (2014).

Supplementary Figure and Figure legends

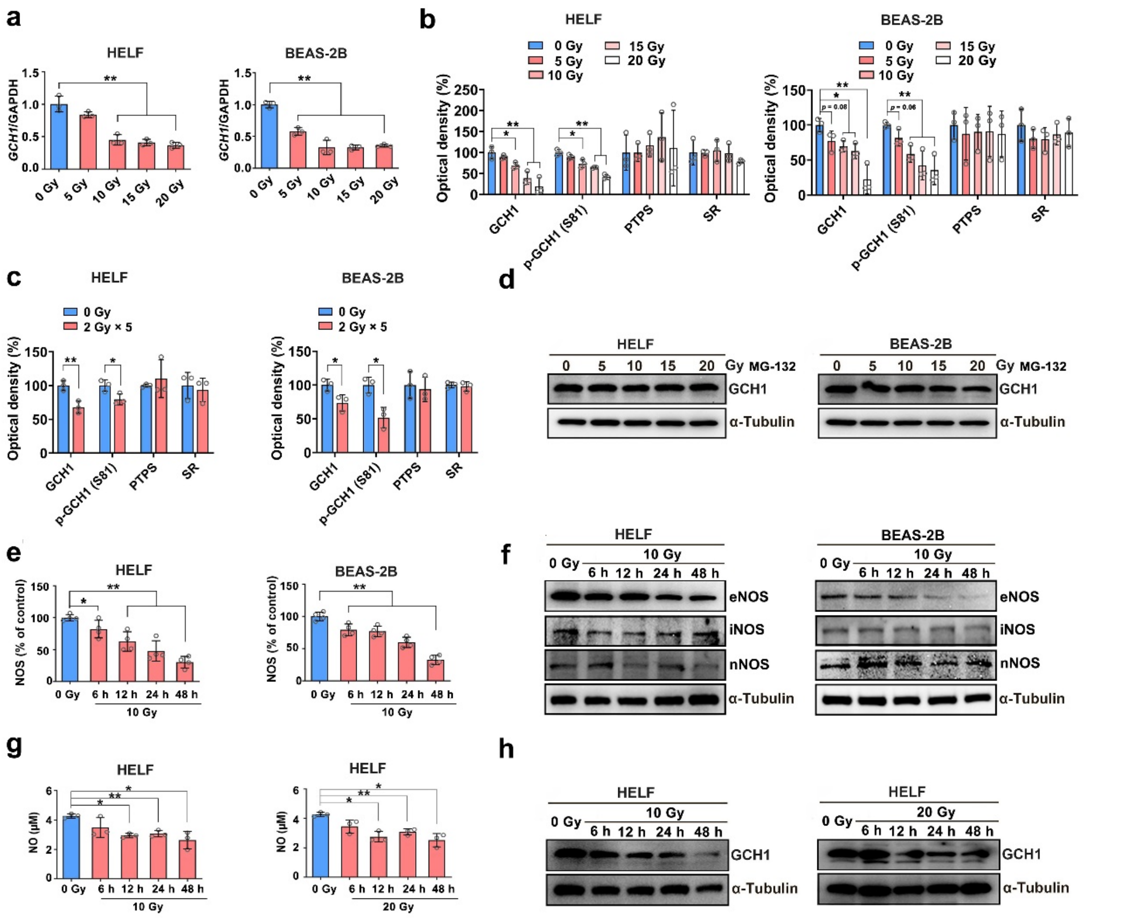

Supplementary Fig. 1. Radiation decreased cellular NO levels, and GCH1-mediated BH4 metabolism reduced ROS levels in pulmonary cells

(a) GCH1 mRNA was detected by qRT–PCR assay in HELF and BEAS-2B cells 24 h after the indicated doses of radiation. (b) Quantitative analyses of GCH1, p-GCH1 (S81), PTPS and SR in HELF and BEAS-2B cells after 0, 5, 10, 15 or 20 Gy irradiation were measured by Image J software. (c) Quantitative analyses of GCH1, p-GCH1 (S81), PTPS and SR in HELF and BEAS-2B cells treated with radiation (10 Gy/5 fractions, 1 fraction per day). (d) HELF and BEAS-2B cells were pre-treated with MG-132 (10 μM) for 4 h and then exposed to the indicated doses of radiation. Twenty-four hours later, Western blotting analysis was

performed to detect GCH1 protein. (e) NOS activity at different time points after irradiation. (f) Western blotting assay was performed to detect eNOS, iNOS and nNOS protein levels. (g) The NO concentration at different time points after irradiation (10 and 20 Gy) was indirectly measured using a Griess assay. (h) GCH1 protein levels at different time points after irradiation (10 and 20 Gy) were measured. * $P < 0.05$ and ** $P < 0.01$ compared with the control group.

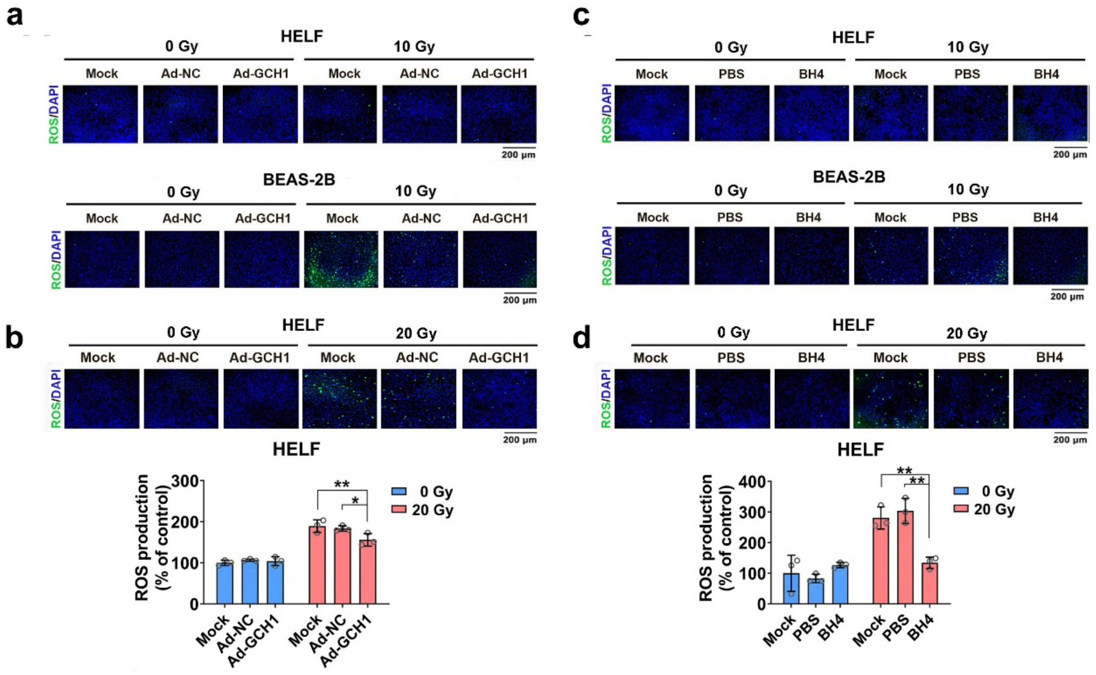

Supplementary Fig. 2. GCH1-mediated BH4 metabolism alleviates ROS levels in irradiated lung cells.

HELF and BEAS-2B cells were infected with Ad-NC or Ad-GCH1 or treated with PBS or BH4 followed by 10 and/or 20 Gy X-ray irradiation. (a-d) The ROS levels were determined using a fluorescence microscope and/or 96-well plate reader. Scale bar = 200 μm. * $P < 0.05$ and ** $P < 0.01$ compared with the control group.

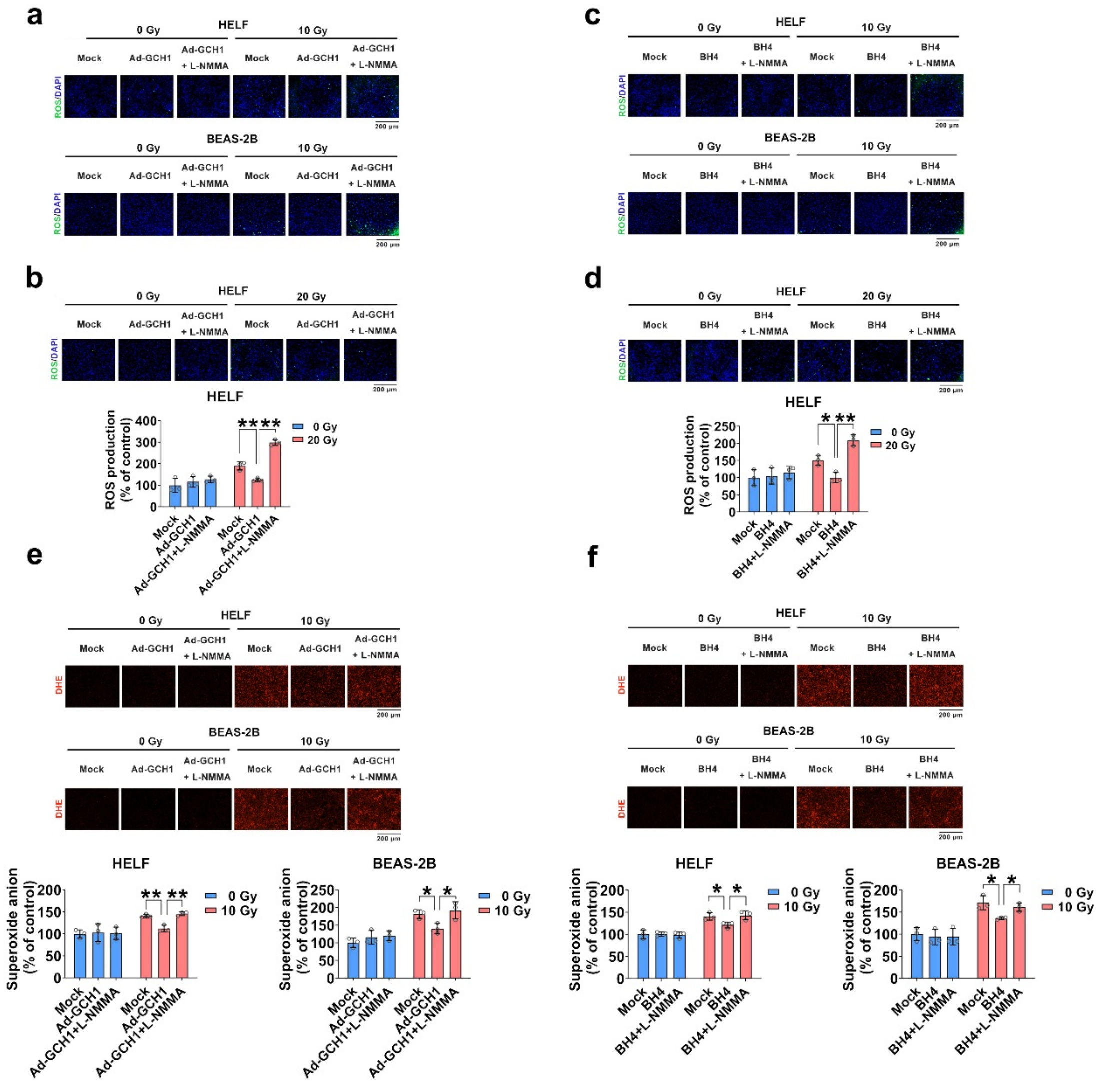

Supplementary Fig. 3. BH4 biosynthesis decreases the level of NOS uncoupling-associated ROS

HELF and BEAS-2B cells were incubated with the NOS inhibitor L-NMMA (200 μM for HELF cells and 100 μM for BEAS-2B cells) for 6 h and then infected with Ad-NC or Ad-GCH1 and PBS or BH4 followed by 10 and/or 20 Gy X-ray irradiation. (a-d) The ROS levels were determined using a fluorescence microscope and/or 96-well plate reader. Scale bar = 200 μm. (e-f) DHE fluorescent probe was used to measure the superoxide anions in HLEF and BEAS-2B cells. Scale bar = 200 μm. HELF and BEAS-2B cells were incubated with another NOS inhibitor L-NAME (100 μM for HELF and BEAS-2B cells) for 6 h and then

infected with Ad-NC or Ad-GCH1 and PBS or BH4 followed by 10 Gy X-ray irradiation. * $P < 0.05$ and ** $P < 0.01$ compared with the control group.

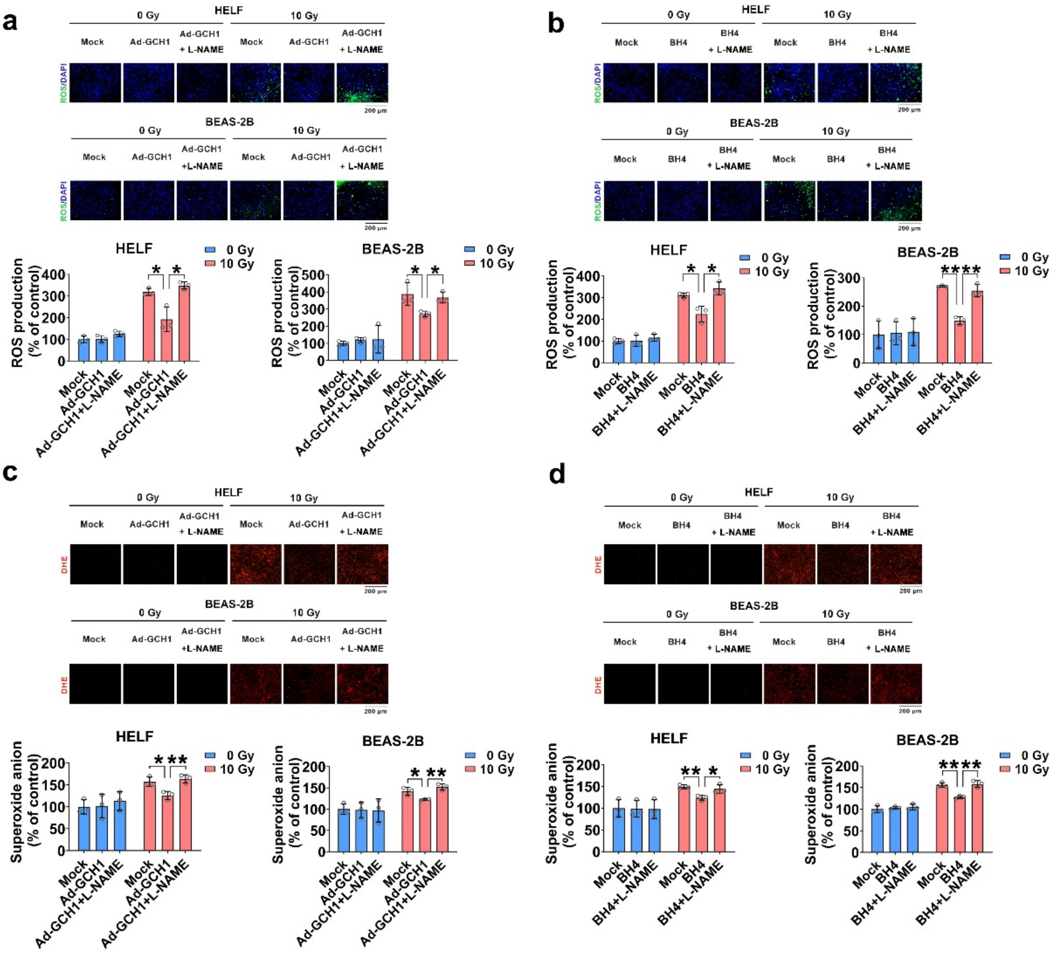

Supplementary Fig. 4. GCH1-mediated BH4 metabolism decreases the level of NOS uncoupling-associated ROS

(a-b) The ROS levels were determined using a fluorescence microscope and/or 96-well plate reader. Scale bar = 200 μm. (c-d) DHE fluorescent probe was used to measure the superoxide anions in HLEF and BEAS-2B cells. Scale bar = 200 μm.* *P* < 0.05 and ** *P* < 0.01 compared with the control group.

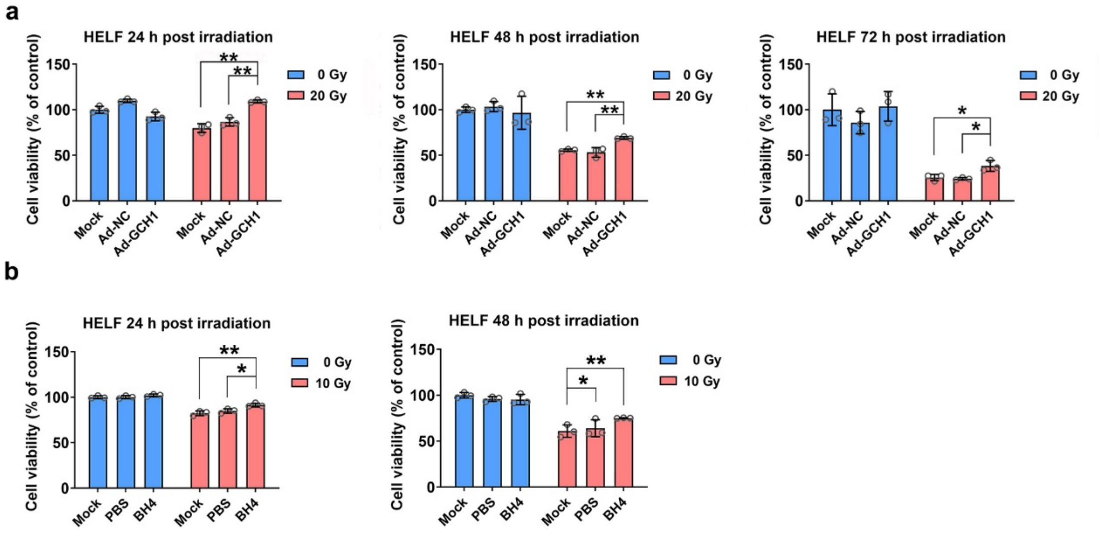

Supplementary Fig. 5. GCH1-mediated BH4 metabolism protects pulmonary cells against radiation-induced damage.

(a) HELF cells were pre-infected with control adenovirus or GCH1 adenovirus and subsequently irradiated. Cell viability was measured by a CCK-8 assay at 24, 48 and 72 h after 20 Gy X-rays. (b) HELF cells were pre-treated with BH4 and subsequently irradiated. Cell viability was measured by a CCK-8 assay at 24, 48 after 10 Gy X-rays. * $P < 0.05$ and ** $P < 0.01$ compared with the control group.

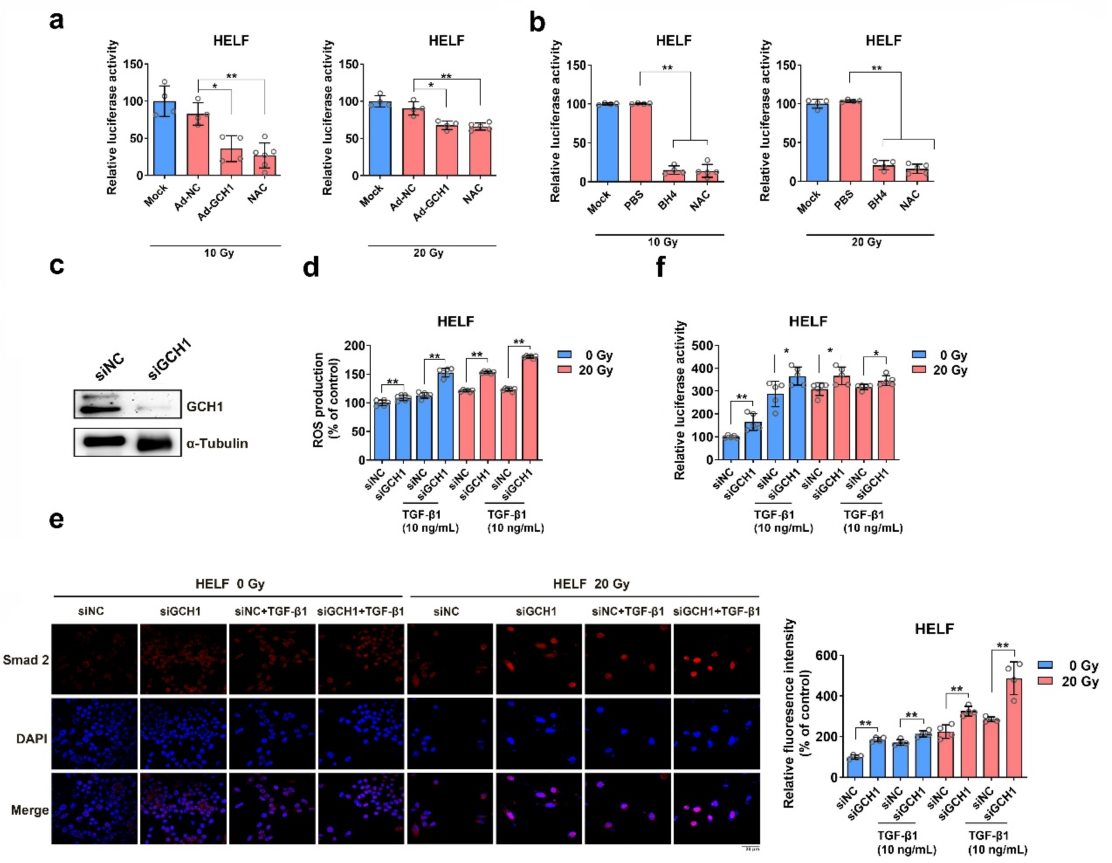

Supplementary Fig. 6. Effect of BH4 anabolism on the fibrotic phenotype of irradiated pulmonary cells.

(a) HELF cells were preincubated with or without NAC before infection with Ad-NC or Ad-GCH1 and subsequently irradiated. Luciferase assay of Smad2/3-responsive luciferase reporter activity after infection with Ad-NC or Ad-GCH1 with irradiation. (b) HELF cells were preincubated with or without NAC before the addition of PBS or BH4 and subsequently irradiated. Luciferase assay of Smad2/3-responsive luciferase reporter activity with BH4 with radiation. HELF cells were transfected with control siRNA (siNC) or *GCH1*-silencing siRNA (siGCH1). (c) Western blotting analysis of GCH1 expression in HELF cells. (d) SiRNA-transfected cells were treated with 10 ng/mL TGF-β1 and/or 20 Gy irradiation, and the cellular ROS levels were determined using a 96-well plate reader. (e) The distribution

of Smad2 in siRNA-transfected cells was detected using immunofluorescence 24 h after administration. (f) Luciferase assay of Smad2/3-responsive luciferase reporter activity after 10 ng/mL TGF-β1 treatment and/or 20 Gy irradiation in siRNA-transfected cells. * $P < 0.05$ and ** $P < 0.01$ compared with the control group.

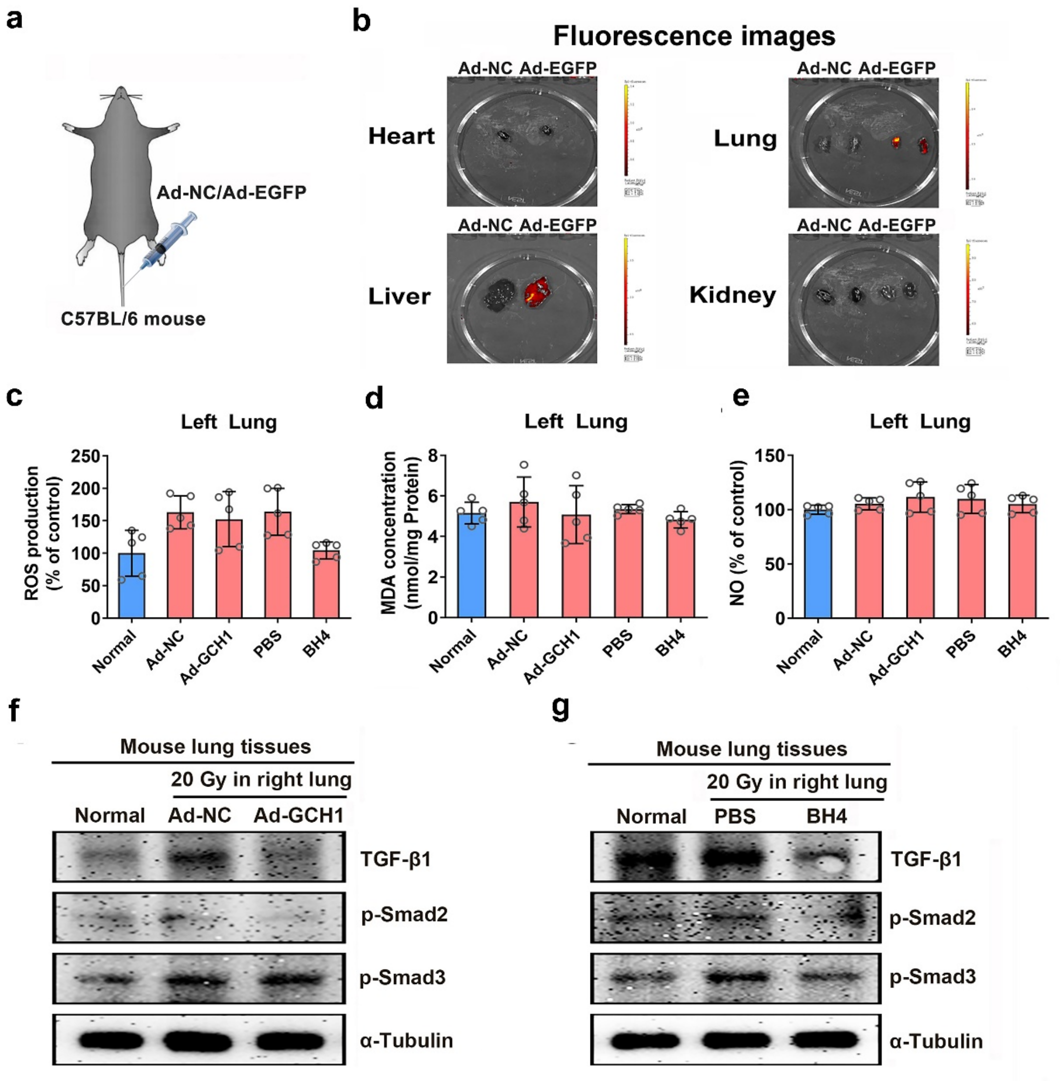

Supplementary Fig. 7. GCH1-mediated BH4 metabolism attenuates acute radiation-induced lung injury in mice.

(a) Experimental scheme for mouse treatment. Mice separately received tail intravenous injection of 100 μL of PBS or Ad-EGFP (1.0×10^{12} vp/mL). (b) The image was visualized by a small animal fluorescence imaging system (Olympus) three days later. (c) Mouse right lung was irradiated with a single dose of 20 Gy X-ray irradiation followed by *in vivo* injection with Ad-NC or Ad-GCH1 and PBS or BH4 (n = 5). Relative ROS levels in the mouse left lung. (d) MDA levels in left lung tissues of mice were determined for the various groups. (e)

NO levels in mouse left lung were detected per group. (f-g) Seven days after irradiation, right lung tissues were lysed and subjected to Western blotting analysis to assess TGF-β1, p-Smad2, and p-Smad3 (S423/425).

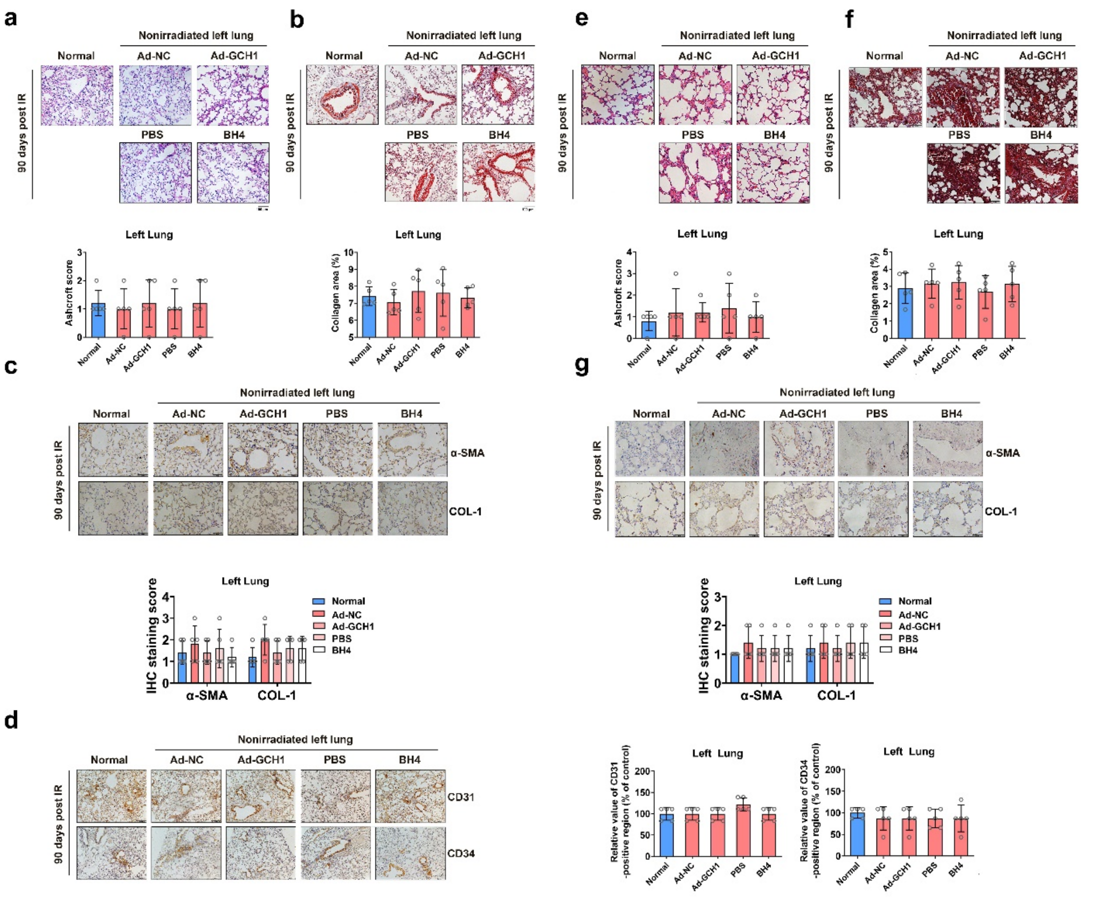

Supplementary Fig. 8. GCH1/BH4 has no effect on the nonirradiated left lung.

Mouse right lung was left without irradiation or irradiated with a single dose of 20 Gy X-ray irradiation followed by *in vivo* injection with Ad-NC or Ad-GCH1 and PBS or BH4 (n = 5). (a) Representative H&E staining of mouse left lung. Scale bar = 100 μm. (b) Representative Masson's trichrome staining of the left lung in mice. Scale bar = 100 μm. (c) Left lung tissues were immunostained with α-SMA and COL-1 and counterstained with haematoxylin. Scale bar = 50 μm. Quantitative analysis of α-SMA- and COL-1-positive cells in slides from nonirradiated mouse lung tissues. (d) CD31 and CD34 levels in left lung tissues of mice. Scale bar = 200 μm. Rat right lung was left without irradiation or irradiated with a single dose of 20 Gy X-ray irradiation followed by *in vivo* injection with Ad-NC or Ad-GCH1 and PBS or BH4 (n = 5). (e) Representative H&E staining of the rat left lung. Scale bar = 100

μm. (f) Representative Masson's trichrome staining of the left lung in rats. Scale bar = 100 μm. (g) Left lung tissues were immunostained with α-SMA and COL-1 and counterstained with haematoxylin. Scale bar = 50 μm. Quantitative analysis of α-SMA- and COL-1-positive cells in slides from nonirradiated rat lung tissues.

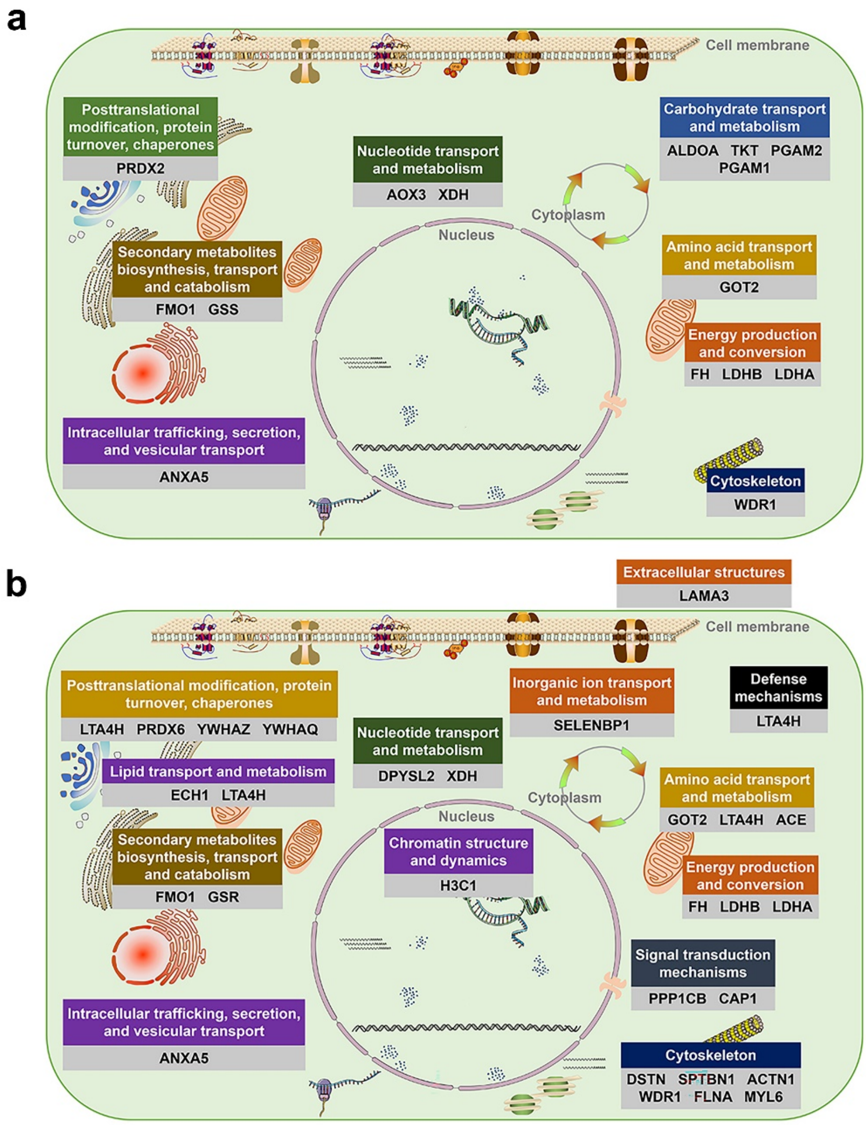

Supplementary Fig. 9. The localization and functional classification of BH4-affected protein S-nitrosylation.

Gene Ontology (GO) analysis was performed to investigate the functional importance of BH4-affected protein S-nitrosylation. Protein S-nitrosylation was enriched in biological pathways related to intracellular trafficking, secretion, vesicular transport, nucleotide transport and metabolism and amino acid transport and metabolism. (a) Proteins with downregulated S-nitrosylation in the 20 Gy vs. 0 Gy group; (b) Proteins with upregulated S-nitrosylation in the BH4 vs. PBS group of mice with 20 Gy radiation.

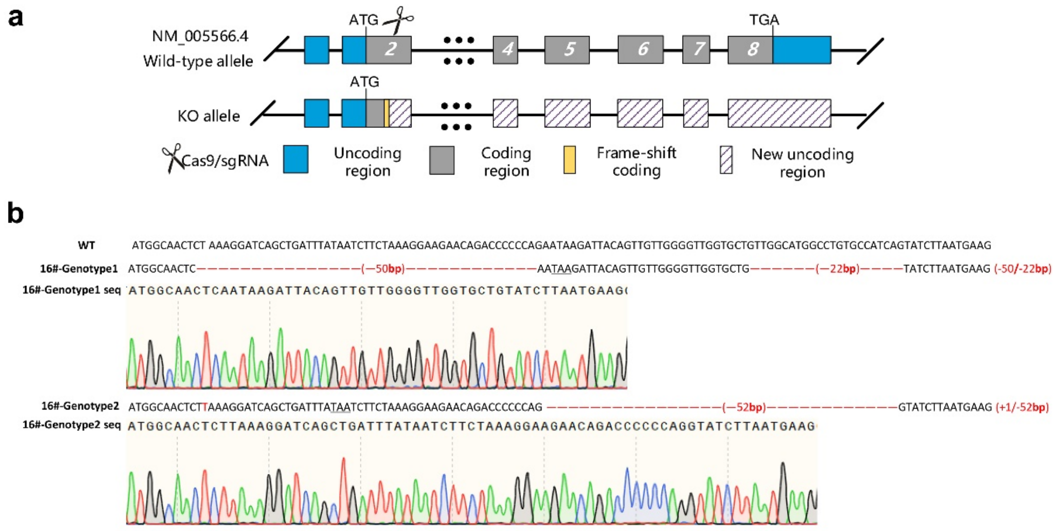

Supplementary Fig. 10. Generation of *LDHA* knockout (KO) BEAS-2B cells using the CRISPR/Cas9 system.

(a) Schematic illustration of the *LDHA* gene structure. According to the structure of the *LDHA* gene, exon 2 or exon 6 of the NM_005566.4 transcript of the *LDHA* gene was selected as the knockout region to design the sgRNA. The double-stranded DNA of the target gene was cut by sgRNA/Cas9, and the base was randomly deleted or inserted by cell nonhomologous terminal junction repair, which caused the reading frame to be framed, forming an early termination codon, and thus could not encode the correct protein. (b) The genomic sequences around the target sites of WT LDHA and *LDHA*-KO in BEAS-2B cells. DNA sequence obtained from PCR fragments of genomic DNA.

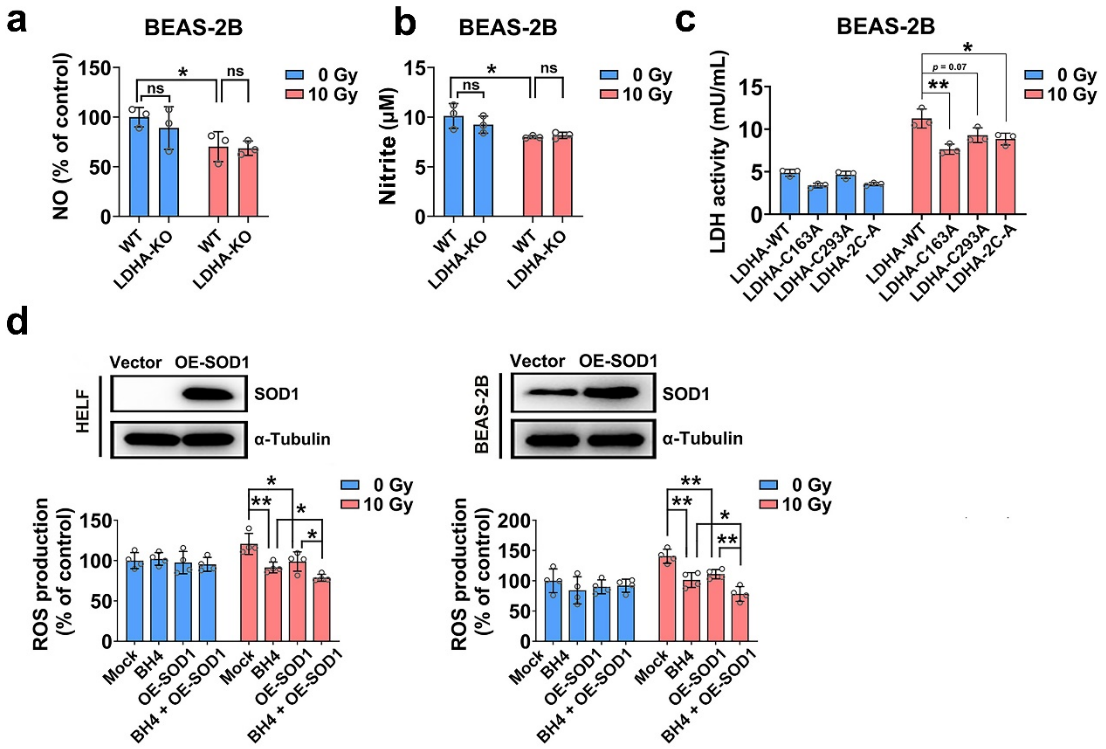

Supplementary Fig. 11. BH4 regulates ROS-generating activity through LDHA S-nitrosylation

(a)The NO concentration in BEAS-2B cells was measured using an NO-sensitive probe and the Griess assay. (b) *LDHA*-KO BEAS-2B cells were transfected with WT LDHA or LDHA mutants followed by 10 Gy X-ray irradiation. And then LDH activity was detected. (c) HELF and (d) BEAS-2B cells were transfected with SOD1 vector, and Western blotting analysis was performed to measure SOD1 protein levels. Cells were treated with BH4 and/or SOD1 plasmid, and then ROS-producing activities were measured with or without radiation. * *P* < 0.05 and ** *P* < 0.01 compared with the control group.
